# Tissue-Specific Effects of Genetic and Epigenetic Variation on Gene Regulation and Splicing

**DOI:** 10.1371/journal.pgen.1004958

**Published:** 2015-01-29

**Authors:** Maria Gutierrez-Arcelus, Halit Ongen, Tuuli Lappalainen, Stephen B. Montgomery, Alfonso Buil, Alisa Yurovsky, Julien Bryois, Ismael Padioleau, Luciana Romano, Alexandra Planchon, Emilie Falconnet, Deborah Bielser, Maryline Gagnebin, Thomas Giger, Christelle Borel, Audrey Letourneau, Periklis Makrythanasis, Michel Guipponi, Corinne Gehrig, Stylianos E. Antonarakis, Emmanouil T. Dermitzakis

**Affiliations:** 1 Department of Genetic Medicine and Development, University of Geneva Medical School, Geneva, Switzerland; 2 Institute of Genetics and Genomics in Geneva (iGE3), Geneva, Switzerland; 3 Swiss Institute of Bioinformatics (SIB), Geneva, Switzerland; 4 Departments of Pathology and Genetics, Stanford University, Stanford, California, United States of America; 5 Center of Excellence in Genomic Medicine Research, KingAbdulaziz University, Jeddah, Saudi Arabia; Perelman School of Medicine, University of Pennsylvania, UNITED STATES

## Abstract

Understanding how genetic variation affects distinct cellular phenotypes, such as gene expression levels, alternative splicing and DNA methylation levels, is essential for better understanding of complex diseases and traits. Furthermore, how inter-individual variation of DNA methylation is associated to gene expression is just starting to be studied. In this study, we use the GenCord cohort of 204 newborn Europeans’ lymphoblastoid cell lines, T-cells and fibroblasts derived from umbilical cords. The samples were previously genotyped for 2.5 million SNPs, mRNA-sequenced, and assayed for methylation levels in 482,421 CpG sites. We observe that methylation sites associated to expression levels are enriched in enhancers, gene bodies and CpG island shores. We show that while the correlation between DNA methylation and gene expression can be positive or negative, it is very consistent across cell-types. However, this epigenetic association to gene expression appears more tissue-specific than the genetic effects on gene expression or DNA methylation (observed in both sharing estimations based on P-values and effect size correlations between cell-types). This predominance of genetic effects can also be reflected by the observation that allele specific expression differences between individuals dominate over tissue-specific effects. Additionally, we discover genetic effects on alternative splicing and interestingly, a large amount of DNA methylation correlating to alternative splicing, both in a tissue-specific manner. The locations of the SNPs and methylation sites involved in these associations highlight the participation of promoter proximal and distant regulatory regions on alternative splicing. Overall, our results provide high-resolution analyses showing how genome sequence variation has a broad effect on cellular phenotypes across cell-types, whereas epigenetic factors provide a secondary layer of variation that is more tissue-specific. Furthermore, the details of how this tissue-specificity may vary across inter-relations of molecular traits, and where these are occurring, can yield further insights into gene regulation and cellular biology as a whole.

## Author Summary

In order to better understand how genetic differences between individuals can cause diseases, it is crucial to understand how genetic variants affect cellular functions in the different tissues that compose the human body. From the umbilical cord of 195 newborn babies, we previously obtained three different cell-types: fibroblasts, T-cells and immortalized B-cells. From every individual in each cell type we measured four features across the genome: 1) genetic differences, 2) DNA methylation, an epigenetic modification of DNA that can affect its functional state, 3) gene expression—the amount of gene activity, 4) alternative splicing—which of the different versions of a gene is manifested. We find thousands of genetic variants of the DNA sequence that affect methylation, gene expression, and splicing. We show that while these genetic effects often affect multiple cell-types, the strength of these effects varies between cell-types. Also epigenetic methylation marks of DNA associate to gene expression and particularly often to splicing. Since abnormalities in gene expression, DNA methylation and alternative splicing are associated to diseases, it is important to continue studying how these traits are inter-related and affected by genetic variation across cell-types.

## Introduction

Understanding how our genome determines the distinct cell-types, tissues and organs that together make a functional human body is essential for better understanding of complex traits and susceptibility to disease. Multiples studies have shown how genetic variation among individuals can affect basic cellular phenotypes, such as gene expression levels [1, 2, 3, 4, 5]. Others have sought to dissect the tissue-specific genetic architecture of gene regulation [6, 7, 8, 9], which has been relevant for better understanding non-coding signals detected by genome wide association studies (GWAS) and complex diseases [10, 11, 12, 13]. Additional studies have also identified genetic variants associated to alternative splicing using microarrays [14, 15, 16, 17, 18]. Furthermore, RNA-seq technology has allowed initial assessments of differential isoform usage associated to genetic variation using distinct approaches in lymphoblastoid cell lines [3, 4, 19]. However, more comprehensive assays in a larger collection of cell-types remain to be done. More recently, studies have also shown the presence of genetic variation affecting DNA methylation levels in several cell-types [20, 21, 22, 23]. Deeper studies of this type will be of great functional value for interpreting the wave of epigenome wide associations studies (EWAS) to come [24].

The role of DNA methylation in gene expression variation is not well understood [25]. Even though it is typically associated to gene silencing [26, 27], recent discoveries have revealed distinct types of participation of DNA methylation in gene regulation. DNA methylation in gene bodies can be positively associated to gene transcription [28, 29]. It can also be a marker of alternative intra-genic promoters [30] and of tissue-specific regulatory elements [31]. Additionally, DNA methylation levels can be affected by transcription factors (TFs) binding at enhancers [32] and others have reported that DNA methylation itself can affect the binding of TFs such as MYC [33]. Moreover, the differential methylation levels found at exon-intron boundaries [34] could indicate that DNA methylation might be involved in alternative splicing. A study found that absence of DNA methylation can promote inclusion of a CD45 exon by allowing CTCF binding and RNA polymerase II pausing [35] and another study found some other cases of DNA methylation sites associated to alternative-splicing in cancer patients [36]. Furthermore, relationships between DNA methylation and gene expression in a population context have been reported to be both positive and negative [20, 22, 23, 36, 37], but they have not been systematically analyzed in high resolution and compared across tissues.

New sequencing technologies, genome-wide assays and comprehensive genome annotation are now offering opportunities to interrogate genome function in multiple individuals at the cellular level. We have previously published the GenCord data of sequenced transcriptomes and assayed methylation levels in three cell-types of a genotyped cohort of 204 individuals [37](see Materials and Methods for a summary). In that study we analyzed the relationships among genetic variation, DNA methylation and gene expression in order to infer the passive and active roles of DNA methylation in gene regulation. We shed light on the context specificity of DNA methylation in gene regulation and on one of the mechanisms by which DNA methylation can have a passive role, being influenced by variant levels of TFs among individuals. In this study we expand our analyses with the objective of addressing the tissue-specificity of the genetic and epigenetic associations to gene expression, and of allele specific expression. Additionally, we measure alternative splicing levels and analyze how it is associated to genetic variation and DNA methylation across cell-types.

## Results

### Genetic effects on gene expression and DNA methylation

**Figure 1:**
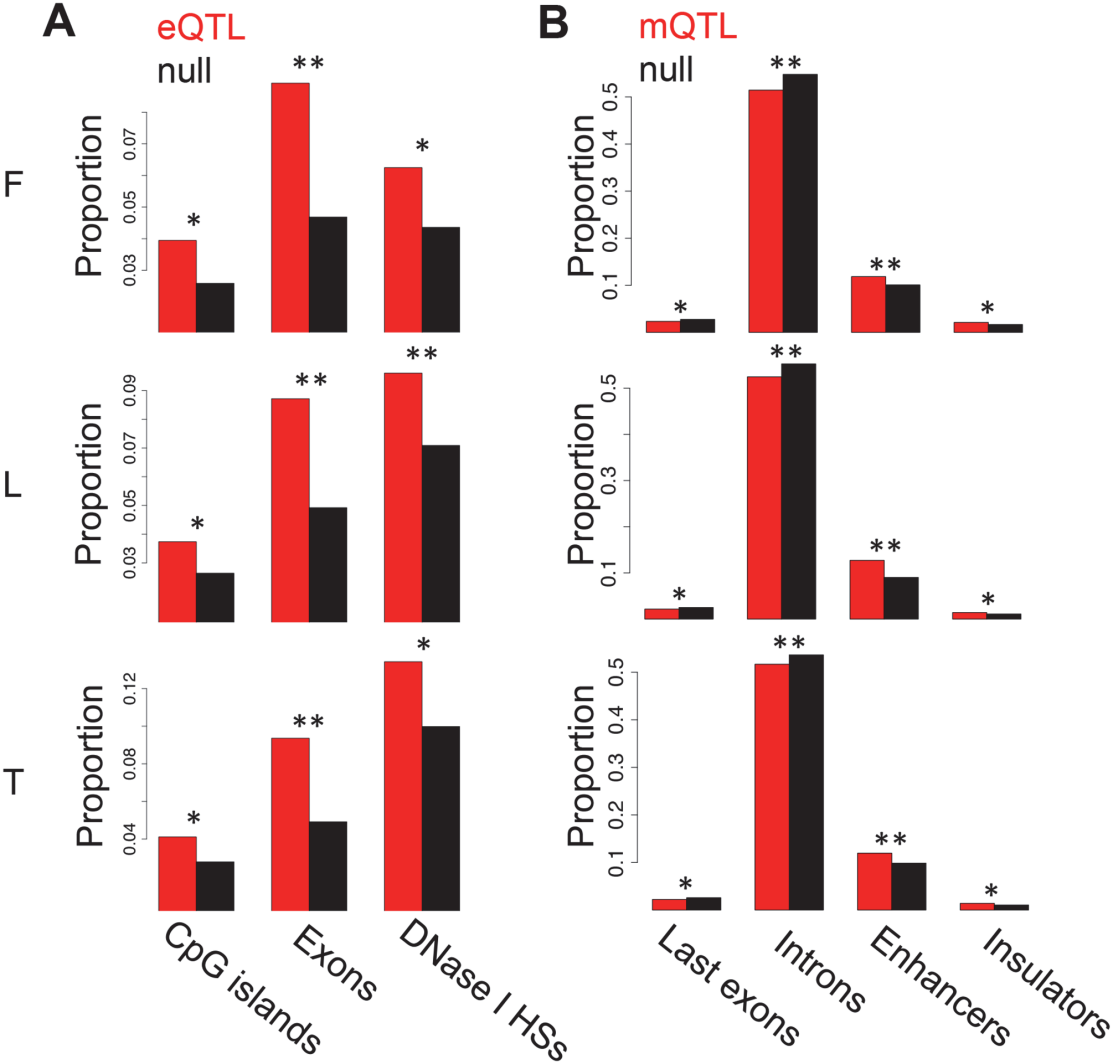
Enrichment of eQTLs and mQTLs in distinct genomic regions. For distinct genomic regions, the proportion of overlapping eQTLs (A) and mQTLs (B) (both in red) was compared to the proportion of overlapping null SNPs (black, see Methods for details). We found significant enrichment of eQTLs in CpG islands, exons and DNase I hypersensitive sites (HSs), and significant enrichment of mQTLs in enhancer and insulator marks, as well as significant depletion in last exons and introns. One star indicates *P* < 0.05, two stars indicate *P* < 5E-04, Fisher’s exact test.

We previously reported the discovery of *cis* associations between genetic variation and gene expression (expressed Quantitative Trait Loci; eQTLs) and between genetic variation and DNA methylation (methylation QTLs; mQTLs) in primary fibroblasts, EBV-transformed lymphoblastoid cell lines (LCLs) and primary T-cells of newborn babies, which are shown in Table 1[37]. Here, we have assessed the level of replication of the LCL eQTLs with those LCL eQTLs reported in a more powered RNA-seq study using older cell-lines from adult individuals [5]. This yields a replication of about 70% based on proportion of true positive from a P-value distribution [38] and effect size comparison (S1 Fig.). In this study we have also analyzed the location of the previously discovered eQTLs and mQTLs. As observed in previous microarray studies, highly significant eQTLs cluster close to the TSS [39]. Additionally, when eQTL genes are classified by whether they are called significant in one, two, or three cell-types, we observe that eQTLs significant in all cell-types tend to be less distant to the TSS than eQTLs significant in two cell-types, and these are less distant than those significant in only one cell-type (S2 Fig.). The same pattern is observed for the LCL eQTLs that were replicated in an independent data set, as well as for a similar analysis that deals better with winner’s curse (S2C-D Fig.). This replicates the patterns observed in previous studies [6, 22], and although some eQTLs may be misclassified due to winner’s curse, this pattern may reflect the importance of distant regulatory elements, such as enhancers, in tissue-specific regulation. Additionally, we also find a large proportion of eQTLs very close to the transcription end site (TES; S3 Fig.), similar to previous observations [4, 40]. Also confirming previous studies with lower resolution arrays [20], highly significant mQTLs are overrepresented close to the interrogated CpG site (*P* < 1.3E-14; S4 Fig.). In all cell-types, we observe that the best eQTLs per gene are significantly enriched in DNase I hypersensitive sites, exons and CpG islands (Fig. 1A; see also S5 Fig.). Also in all cell-types, mQTLs are significantly enriched in enhancers and insulators, and depleted in last exons and introns (Fig. 1B; see also S6 Fig.). Several of these QTLs involve SNPs that have been reported to be associated to various diseases and traits according to literature of genome wide association studies [41] (GWAS)- 8, 3 and 4 eQTLs, and 32, 51 and 74 mQTLs, in fibroblasts, LCLs and T-cells, respectively—although this enrichment is not significant and does not necessarily imply causal relationship between these eQTLs or mQTLs and disease. In conclusion, genetic variants affecting gene expression and DNA methylation levels often overlaps with functional genomic elements. This also indicates that the DNA sequence variation greatly influences the level of methylation. The genetic variants affecting DNA methylation are predominantly located in distant regulatory regions, as shown here, or in non-CpG island promoters as shown before [37], rather than inside genes. These results are compatible with the observations of differential methylation across tissues being predominantly located distant to transcription start sites [31], and enriched for inter-individual methylation variation associated to genetic variation [37].

**Table 1:** Summary of associations and allele-specific expression analyses in GenCord.

	**Test**	**Samples**	**Window size**	**Phenotype**	**FDR**	**Nominal P-value**	**F**	**L**	**T**
eQTLs	genotypes and expression	183(F)	1Mb	genes	10%	2.2E-05(F); 3.2E-05(L); 1.8E-05(T)	2433	3372	2115
		185(L)							
		186(T)							
mQTLs	genotypes and methylation	107(F)	5kb	methylation sites	10%	4.4E-04(F); 7.9E-04(L); 1.3E-3(T)	14189	22411	32318
		111(L)							
		66(T)							
eQTMs	methylation and expression	110(F)	50kb	genes	10%	7.6E-05(F); 7E-04(L); 6.9E-04(T)	596	3680	3838
		118(L)							
		66(T)							
asQTLs	genotypes and alt. splicing	183(F)	1Mb	genes	10%	Permutation per exon-exon link	382	527	380
		185(L)							
		186(T)							
asQTMs	methylation and alt. splicing	110(F)	50kb	genes	10%	Permutation per exon-exon link	4602	5663	81
		118(L)							
		66(T)							
ASE	allelic imbalance	178(F)	-	assayable heterozygous sites	20%*	0.005	7162	8211	7747
		178(L)							
		180(T)							

Number of significant genes, methylation sites or assayable heterozygous sites in fibroblasts (F), LCLs (L) and T-cells (T). The association analyses on eQTL, mQTLs and eQTMs were previously reported [37].

* FDR calculated as number of expected over number of observed based on the nominal P-value threshold.

We next sought to study the degree of tissue specificity of eQTLs and mQTLs. Of the significant associations at 10% FDR, 47–60% of eQTL genes and 48–66% of mQTL CpG sites are found in at least two cell-types (S7–S8 Figs.). We then used a more continuous measure of tissue sharing percentage called the π1 statistic [38]. When assessing what proportion of the effects in a first cell-type are shared with a second cell-type, this statistic estimates the proportion of true positives from the full P-value distribution of SNP-exon or SNP-CpG pairs in the second cell-type, for the pairs that are significant in the first cell-type. LCLs and T-cells share a larger proportion of genetic effects between them (π_1_ = 0.52–0.59 in eQTLs and 0.75–0.80 in mQTLs) than each with fibroblasts (π_1_ = 0.40–0.47 in eQTLs and 0.46–0.58 in mQTLs; Table 2), as expected given their closer developmental origin.

**Table 2:** π_1_ statistic representing fraction of effects shared between cell-types. significant in—π_1_ of P-value distribution in

	**F-L**	**F-T**	**L-F**	**L-T**	**T-F**	**T-L**
**eQTLs**	0.47	0.42	0.40	0.52	0.45	0.59
**mQTLs**	0.57	0.58	0.54	0.80	0.46	0.75
**eQTMs**	0.42	0.25	0.20	0.21	0.13	0.25
**asQTLs**	0.64	0.63	0.58	0.76	0.62	0.81
**asQTMs**	0.66	0.002	0.46	0.003	0.47	0.75
ASE*	0.46	0.50	0.47	0.51	0.47	0.61

* π1 using 30 read cut-off and sampling, see S2 Table for 16 read cut-off.

**Figure 2:**
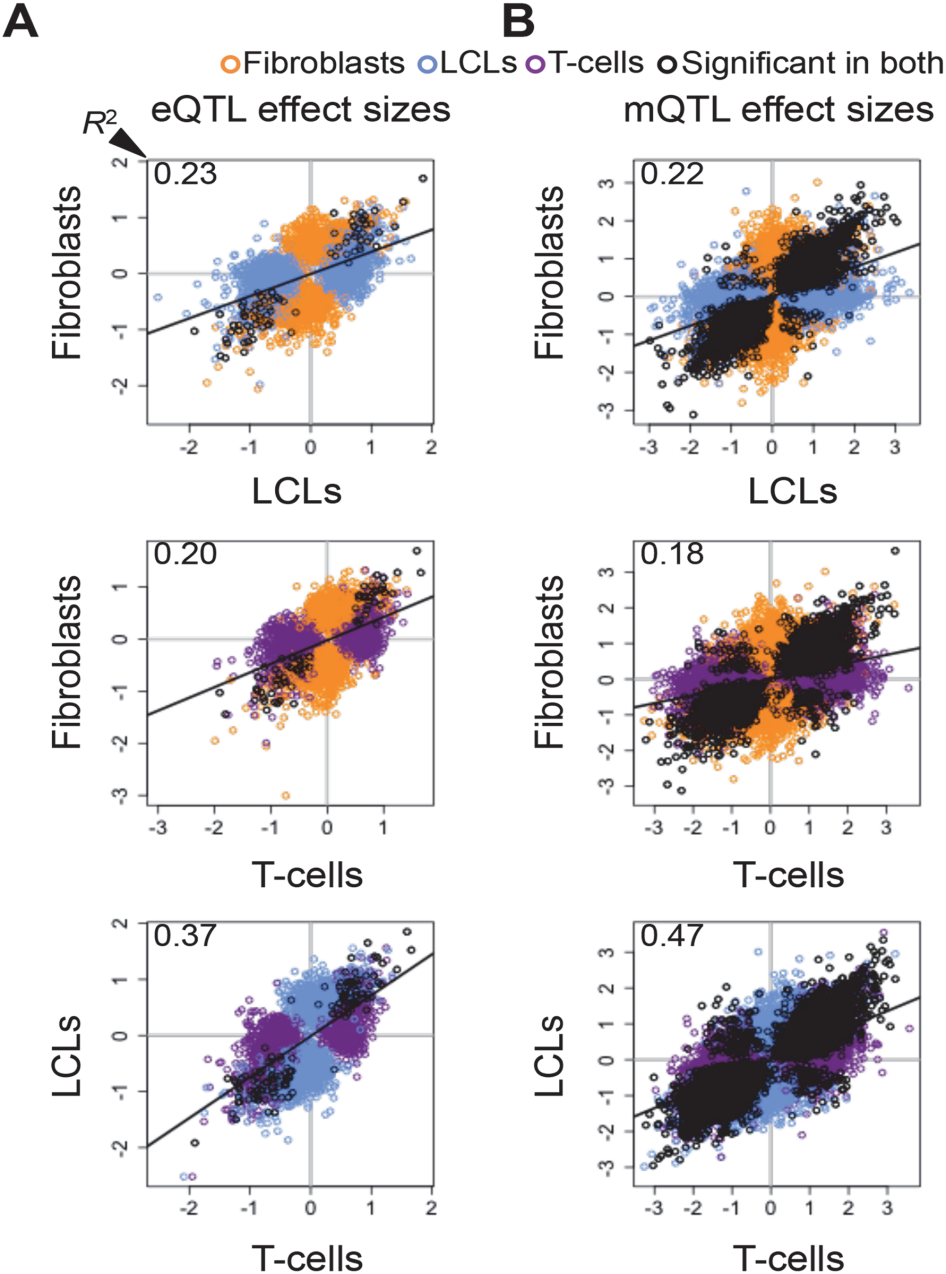
Cell-type specific genetic effects on gene expression and DNA methylation. Effect sizes of expression Quantitative Trait Locus (eQTL) (A) and methylation QTLs (mQTL) (B) unions for each pair of cell-types. Effect size is measured as the difference in medians of scaled expression or scaled methylation level (β-value) between heterozygous individuals and the homozygous individuals for the major allele. Hence, effect sizes are quantified in terms of number of expression or methylation standard deviations changed by an allele modification for eQTLs and mQTLs, respectively. Effect sizes are significantly correlated among all cell-types (Pearson’s correlation *P* < 2.2E-16). Coefficient of determination *R* ^2^, reflecting the proportion of effect size variance in one cell-type explained by the other cell-type, is shown in the top left corner of each plot (S1 Table). SNP-exon pairs or SNP-CpG pairs that are significant in both tissues at 10% FDR are depicted in black, whereas associations significant in only one of the two cell-types compared are in orange (fibroblasts), blue (LCLs) and purple (T-cells). The *R* ^2^ and the spread of the scatter plots show there is a large amount of tissue-specific genetic effects on gene expression and DNA methylation.

To further dissect tissue specificity of genetic effects we compared effect sizes of eQTLs and mQTLs between cell-types. We measured effect sizes as the scaled expression level or scaled methylation level difference between the medians of heterozygous individuals and the homozygous individuals for the major allele (S9–S11 Figs.). Hence, effect sizes are quantified in terms of number of expression or methylation level standard deviations for eQTLs and mQTLs, respectively. The spread of the plots in Fig. 2A-B and the proportion of effect size variance in one cell-type explained by the other (*R* ^2^) show that while effect sizes are significantly correlated between cell-types (all *P*-values < 2.2E-16), they vary substantially when looking at the union of eQTLs or mQTLs between any two cell-types (*R* ^2^ = 0.20–0.37 and 0.18–0.47, respectively; S1 Table). Effect sizes can also vary when looking only at the set of eQTLs or mQTLs significant in both cell-types in each pair wise comparison (*R* ^2^ = 0.88–0.92 and 0.70–0.79, respectively). Interestingly, we found no strong difference in effect sizes between eQTLs or mQTLs significant in multiple cell-types and those significant in only one cell-type, except in fibroblasts, where eQTLs and mQTLs found significant in more than one cell-type have larger effect sizes than significant in only one cell-type (S12 Fig.). Overall, our results show that genetic effects on both gene expression and DNA methylation have a considerable fraction of shared effects between cell-types and recapitulate cell-lineage relatedness but there is also substantial variability among tissues in effects size even among shared QTLs.

### Allele-specific expression

**Figure 3:**
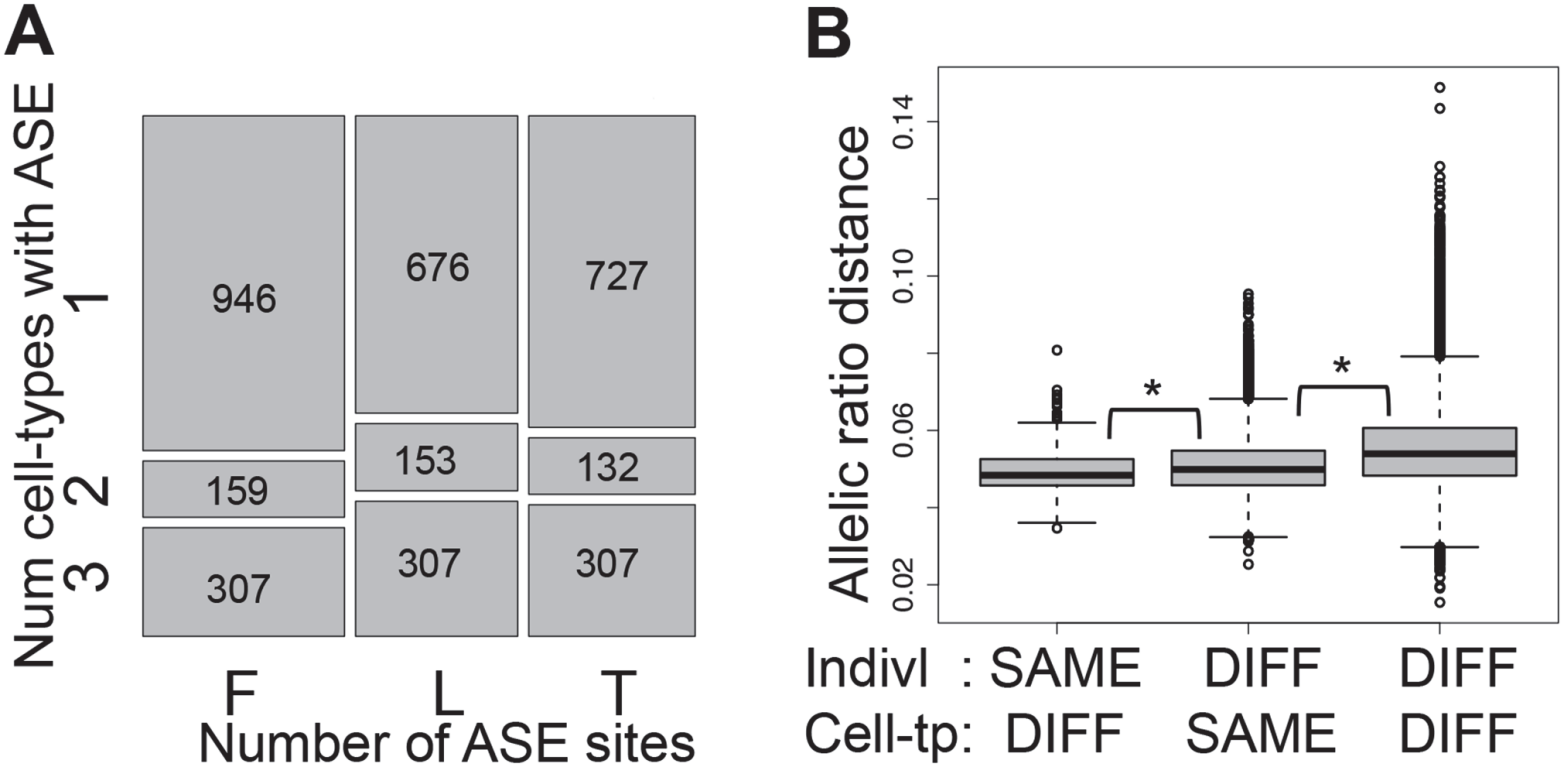
Allele-specific expression cell-type and individual effects. (A) Overlap of Allele-Specific Expression (ASE) across cell-types illustrated by the relative amount of ASE sites (*P* < 0.005) in each cell-type (x-axis) found in one, two or three cell-types within each individual (y-axis), requiring at least 30 reads per site and further sampling to exactly 30 reads. Number of ASE sites falling in each category is indicated in the squares. F, L and T stand for fibroblasts, LCLs and T-cells, respectively. 33–40% of the assayable heterozygous sites in the three cell-types of an individual are in ASE in at least two cell-types (see also S13E Fig.). (B) Distributions of allelic ratio distance (see Materials and Methods) between samples of different (DIFF) or same cell-types (Cell-tp) or individual (Indivl). All the pair wise differences between distributions have *P* < 2.2E-16 (Wilcoxon test). Allelic ratio distances between two cell-types of an individual are smaller than those between two individuals within one cell-type, and these are smaller than those between different individuals and different cell-types. This indicates a strong genetic load at the individual level, but also an important cell-type specific effect.

Several studies have assessed allele specific expression (ASE) of genes as a complementary approach to analyze cis-regulatory variation and to understand its effect on coding variation and disease [3, 4, 42]. In our past study, we used allelic imbalance measures, in assayable heterozygous sites, to show that allele specific expression is predominantly driven by genetic regulatory variation, finding no significant evidence for being driven by DNA methylation [37]. However, there are no studies that have globally looked at ASE in several cell-types of the same individuals to assay the degree of allele specific expression and its sharing among cell-types. Therefore, in this study we have analyzed allele specific expression through binomial testing of the allelic ratio of reads mapping to the assayable heterozygous sites in each individual, after applying stringent criteria for site inclusion and testing (see Methods). Using sites covered by at least 30 reads and sampling additional reads to exactly 30 reads in order to avoid bias from differential coverage, the genes we ended up analyzing are of relatively high expression levels. We tested a median of 1748 heterozygote sites per sample, of which a median of 41 (2.4%) show significant ASE (*P* < 0.005, 20% FDR, Table 1). Of the ASE signals, 33–40% are significant in two or more cell-types of the same individual (Fig. 3A), with π1 values of 0.46–0.61 (Table 2), showing slightly increased sharing between T-cells and LCLs. (see S13 Fig. and S2 Table for results with 16 read cut-off). It is important to keep in mind that in this and other studies, all quantitative analyses of the extent of tissue sharing of eQTL, ASE and other features are to some degree dependent on the specific study design and analysis methods. Thus, they should be interpreted in their specific context.

We then calculated allelic ratio distances between all sample pairs (individual—cell type) as the median of absolute REFERENCE/TOTAL ratio differences of all the shared heterozygous sites covered by > = 40 reads in both samples, weighted by the total number of reads covering the site (see Methods for more details). Genome-wide allelic ratio distances across cell-types and individuals show that the smallest allelic ratio distances are between cell-types within the same individual, whereas the distances between the same cell-type of different individuals are larger (*P* < 2.2E-16, Fig. 3B). This indicates that ASE reflects regulatory effects of the genome that are shared between the cell-types within an individual rather than gene expression levels that are characteristic to each tissue. However, tissue-specificity of regulatory effects is also important: two individuals are closer to each other when comparing the same cell-type than different cell-types (*P* < 2.2E-16, Fig. 3B). In this analysis the allelic ratio distance between two individuals may be genetically driven based on the genotype and phasing of the causal regulatory variant(s). Overall, these results show that although there are many cell-type specific effects on ASE, the genetic profile of each individual contributes more to the variance of allelic imbalance than cell-type specificity.

### DNA methylation and gene expression

**Figure 4:**
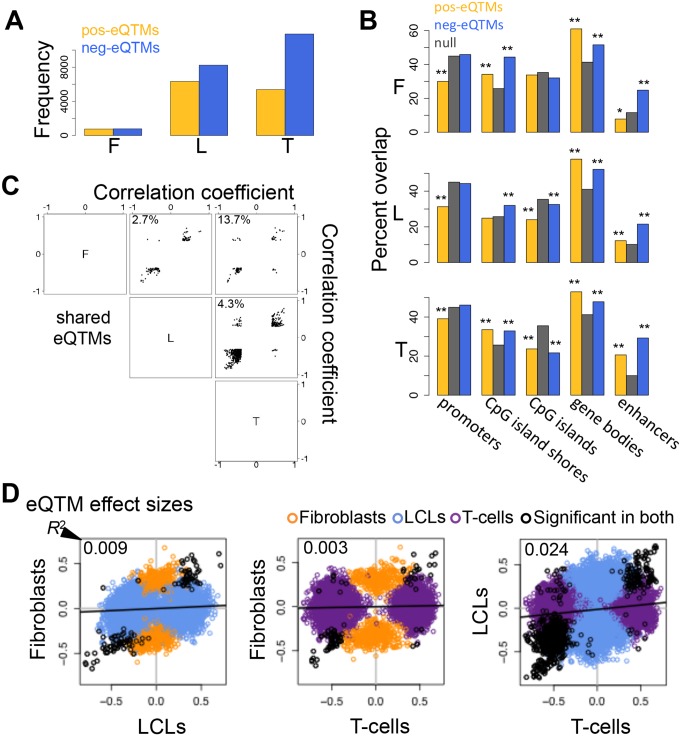
Properties of DNA methylation when associated to gene expression. (A) Number of all significant positive (pos-eQTMs) and negative (neg-eQTMs) correlations between DNA methylation and gene expression in fibroblasts (F), LCLs (L) and T-cells (T). A larger number of neg-eQTMs is found in all three cell-types, but an important part is composed of pos-eQTMs. (B) Percent of pos-eQTMs, neg-eQTMs and null sites that overlap with promoters, CpG island shores, CpG islands, gene bodies and enhancers. Pos-eQTMs are significantly depleted in promoter proximal regions in all cell-types. In most cases both positive and negative eQTMs are enriched for CpG island shores, gene bodies and enhancers, and depleted for CpG islands. One star indicates *P* < 0.05, two stars indicate *P* < 5E-04, Fisher’s exact test. (C) Correlation coefficients of eQTMs significant in both cell-types compared for all pair wise combinations of the three cell-types are plotted. The percentage of discordant cases (associations with opposite sign between any pair of cell-types) is indicated in the top left corner of each panel. Most eQTMs that are significant in any pair of cell-types have the same sign of association. (D) eQTM effect sizes are measured as the slope of the linear regression of expression given methylation on scaled values, and compared between cell-types for union of eQTMs. Black dots are significant eQTMs (same CpG-exon pair) in both the cell-types compared; orange, blue and purple dots are eQTMs significant only in fibroblast, LCL and T-cell, respectively, within the pair compared. Coefficient of determination *R* ^2^, reflecting the proportion of effect size variance in one cell-type explained by the other cell-type, is shown in the top left corner of each plot (S1 Table). This shows that there is a large amount of tissue-specific effects for correlations between DNA methylation and gene expression.

In our earlier study, we reported the significant associations between DNA methylation and gene expression (expression Quantitative Trait Methylation, eQTMs; Table 1), and we observed that these associations were both positive (pos) and negative (neg)[37]. For the sake of clarity we plot here the frequency of each, pos-eQTMs and neg-eQTMs (Fig. 4A), with neg-eQTMs composing 69%, 57% and 51% in T-cells, LCLs and fibroblasts, respectively. The reason for a smaller number of associations in fibroblasts remains unknown and one can hypothesize biological reasons (increased immune system plasticity so higher variation in LCL and T-cell methylation) or technical such as passage effects that are different among the cell types (gene expression and DNA methylation were measured at different passage numbers in fibroblasts and LCLs, but at the same passage number in T-cells). Furthermore, we previously assessed the context specificity of DNA methylation in gene regulation by identifying the genomic regions in which there was a differential proportion of pos-eQTMs versus neg-eQTMs [37]. Here we assess the enrichment of each type of eQTM separately in distinct genomic regions, and we then study their replication across cell-types and their tissue-specificity. As shown in Fig. 4B, in all cell-types there is a significant depletion for pos-eQTMs in promoter proximal regions, similar to previous findings in cancer patients [36]. In most cell-types, we find a significant enrichment for both pos and neg-eQTMs in CpG island shores, gene bodies and enhancers, whereas we observe a significant depletion in CpG islands (Fig. 4B; S3 Table). In conclusion, the methylation sites involved in associations to gene expression often overlap regulatory elements, but in contrast to methylation associated to genetic variation, they also highly overlap gene bodies, which could reflect different roles of DNA methylation in gene regulation.

The vast majority of overlapping (significant in both cell-types compared) eQTMs (86.3–97.3%) have the same sign of correlation in different cell-types (Fig. 4C). We manually checked the 13.7% discordant associations between fibroblasts and T-cells (S4 Table), and discovered that most of them are highly likely to be true positives and could represent quantification of different transcripts (isoforms) that are correlated to methylation positively in one cell-type and negatively in the other. Indeed, there is a high level of differential isoform use between fibroblasts and T-cells in general and more strongly so for the isoforms involving the exons of the discordant eQTMs (see Materials and Methods). However, most eQTMs do not present opposite associations, so the discordant cases could also be due to other tissue-specific context differences in those loci and a small degree of false positives.

Looking at the overlap of eQTMs across cell-types (S14 Fig.), ~44% of genes with eQTMs are observed in two or more cell-types (fibroblasts present a larger percentage of overlap, 63%, probably due to the small number of associations detected in that cell-type). However, most of the eQTM genes overlapping among cell-types involve different CpG sites, hence π1 better reflects the fraction of CpG-exon associations shared: 0.13–0.42 (Table 2). Furthermore, we calculated effect sizes for eQTMs, by scaling the methylation and expression data and estimating the linear regression slope of methylation on expression (S15–S16 Figs.), and compared them across cell-types. Effect size variability between cell-types in eQTMs is much higher than that of eQTLs and mQTLs, reflected by the low *R* ^2^ values that range from 0.003–0.024 for all pair-wise unions of eQTMs (Fig. 4D, S1 Table). Moreover, when looking at the eQTMs significant in both cell-types compared, effect sizes vary much less, with 55–87% of the effect size variance in one cell-type explained by the other. In sum, associations between methylation and expression are replicated across cell-types at consistent directions (positive and negative, when significant in each pair of cell-types compared) and their effect size variability (as well as the π_1_) indicates a higher degree of tissue-specificity than genetic effects.

### Associations to alternative splicing

**Figure 5:**
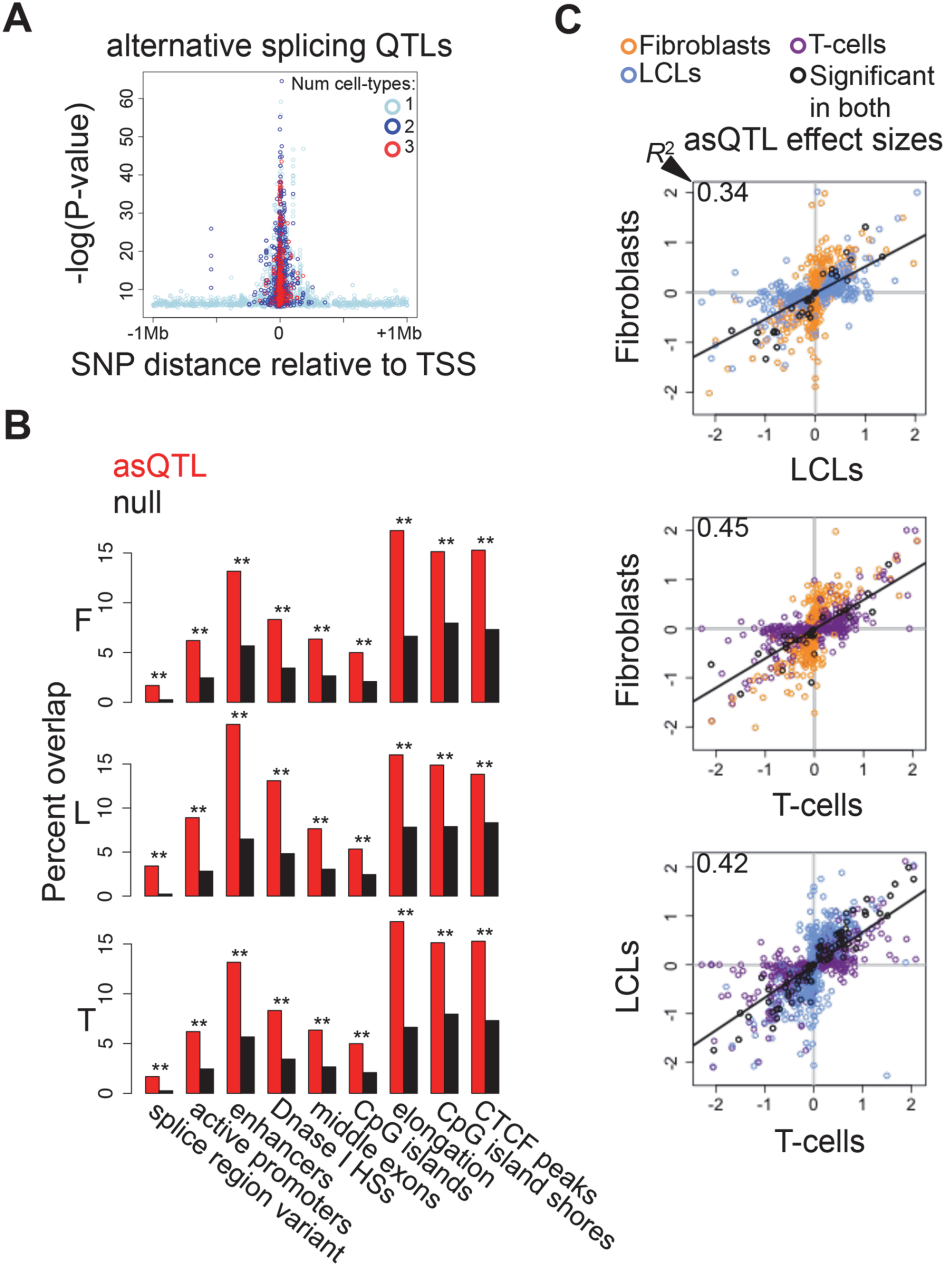
Genetic effects on alternative splicing. (A) Highly significant associations between genetic variation and alternative splicing (asQTLs) cluster close to the transcription start site (TSS). The top asQTL per exon-exon link is plotted showing its relative distance to the TSS by the level of significance—log(P-value). Additionally, we observe that associations called significant in three cell-types are closer to the TSS than associations significant in two cell-types and these are closer than associations significant in only one cell-type. A great part of the genetic control on splicing appears to occur in promoter proximal regions, and cell-type specific distant asQTLs could reflect that distant regulatory elements are involved in tissue-specific splicing. (B) For distinct genomic regions, the percent of overlapping asQTLs (top SNP per link) in comparison to the proportion of overlapping null SNPs in fibroblasts (F), LCLs (L) and T-cells (T). We found significant enrichment of asQTLs in splice region variants, active promoters, enhancers, DNase I hypersensitive sites (HSs), middle exons, CpG islands, elongation marks, CpG island shores and CTCF peaks (see also S5 Table). One star indicates *P* < 0.05, two stars indicate *P* < 5E-04, Fisher’s exact test. (C) Effect sizes of the union of asQTLs (best SNP per exon-exon link) for each pair of cell-types are plotted. Effect size is measured as the difference in medians of scaled alternative splicing levels between heterozygous individuals and the homozygous individuals for the major allele. Hence, effect sizes are quantified in terms of number of standard deviations on the alternative splicing levels modified by an allele change. Black dots depict associations significant in both cell-types compared; orange, blue and purple dots are associations significant in only fibroblasts, LCLs and T-cells, respectively, within the pair compared. Coefficient of determination R^2^, reflecting the proportion of effect size variance in one cell-type explained by the other cell-type, is shown in the top left corner of each plot (S1 Table).

We then analyzed alternative splicing and how it is associated to genetic variation and DNA methylation across cell-types. We measured alternative splicing levels using the algorithm *Altrans* (Ongen H., Dermitzakis E.T., *submitted*) that is based on quantifying exon-exon links between paired reads (see Materials and Methods). To identify associations between SNPs and alternative splicing (asQTL, alternative splicing Quantitative Trait Locus), we calculated the Spearman Rank Correlation between alternative splicing levels and SNP genotypes within 1 Mb of TSSs. From the 4,991–5,853 tested genes, at 10% FDR we find 382, 527 and 380 genes with significant asQTLs in fibroblasts, LCLs and T-cells, respectively (Table 1, S1 Dataset). Based on the π_1_ statistic estimation, about 66% of the LCL asQTLs are replicated in the more powered study of Geuvadis [5] (S17A Fig.). The spearman rank correlation coefficient of our LCL eQTL effect sizes between each dataset is 0.6 (S17B Fig.). We think differences such as split mapping (that was performed in the Geuvadis project only) may account for the slightly lower replication we observe in asQTLs compared to eQTLs. Similar to patterns observed for eQTLs, highly significant asQTLs tend to cluster close to the TSS and at the TES, and the number of cell-types in which an asQTL is significant is associated with distance to the TSS (Fig. 5A, S18 Fig.). By analyzing the best SNP per exon-exon link, we find that asQTLs are significantly enriched for distinct types of regulatory regions—including active promoters, enhancers, DNase I hypersensitive sites and CTCF peaks—as well for middle exons, CpG islands, CpG island shores, elongation marks and very strongly for splice region variants (Fig. 5B; S5 Table). The distance between asQTL SNPs and TSSs is significantly smaller than that observed for eQTLs (all P < 1.6E-19, Wilcoxon test). Additionally, we observed a 1.9 to 4-fold enrichment of asQTLs overlapping GWAS SNPs, although this enrichment is marginally (P = 0.026, fibroblasts) or not significant (LCLs and T-cells, S5 Table). Overall, these results suggest the genetic control on splicing occurs in a wide variety of places within (20–30%) and outside (70–80%) of the gene, with a significant part occurring at distant regulatory regions, which are likely to be involved in tissue-specific alternative splicing.

We next sought to study the degree of tissue-specificity of asQTLs. We observe that 29–44% of asQTL genes are significant in at least two cell-types (S19A Fig.). Analysis of π_1_ on specific SNP-link associations indicates a comparable amount of tissue specificity in asQTLs compared to eQTLs and mQTLs, estimating 58% to 81% of sharing between cell-types (Table 2). Additionally, we calculated the effect sizes of asQTLs taking the same approach as in the other traits (difference in medians of scaled alternative splicing levels between heterozygous and homozygous for the major allele). Comparing effect sizes across cell-types reveals a picture in which 34–46% of the effect size variance of one cell-type is explained by the other when taking the union of asQTLs (Fig. 5C; S1 Table; S20 Fig.). As expected, *R* ^2^ substantially increases when looking solely at the associations reported significant in both cell-types analyzed, ranging from 0.70–0.89.

**Figure 6:**
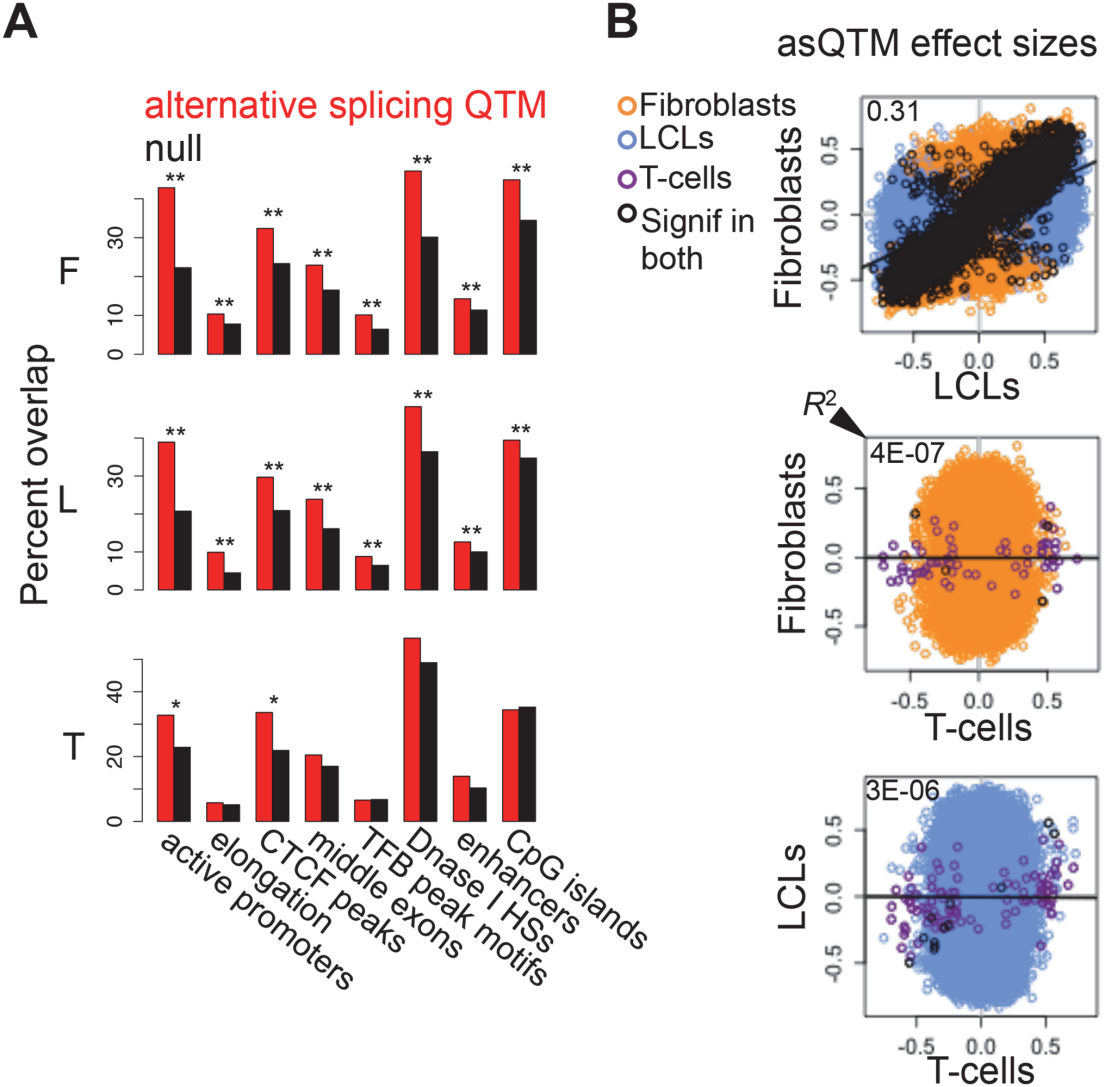
Associations between DNA methylation and alternative splicing. (A) Methylation sites associated to alternative splicing (asQTMs) are significantly enriched for active promoters, elongation marks, CTCF peaks, middle exons, transcription factor binding (TFB) peak motifs, DNase I hypersensitive sites (HSs) and enhancer marks; as shown by the higher percentage of overlap in observed asQTMs compared to null sites. One star indicates *P* < 0.05, two stars indicate *P* < 5E-04, Fisher’s exact test (see also S6 Table). For T-cells there was no ChIP-seq data available at the time of analysis so the data of an LCL was used instead (see Materials and Methods). (B) Effect sizes of the union of asQTMs (best methylation site per exon-exon link) for each pair of cell-types are plotted. Effect size is measured as the slope of the linear regression of alternative splicing levels given methylation levels on scaled values. Hence, effect sizes are quantified in terms of number of alternative splicing standard deviations changed by every methylation standard deviation change. Black dots depict associations significant in both cell-types compared; orange, blue and purple dots are associations significant in only fibroblasts, LCLs and T-cells, respectively, within each pair compared. Coefficient of determination R^2^, reflecting the proportion of effect size variance in one cell-type explained by the other cell-type, is shown in the top left corner of each plot (S1 Table). Given lower sample size in T-cells, there are many less significant associations and hence, it is difficult to assess cell-type specificity between this cell-type and the rest. Fibroblasts and LCLs show a significant fraction of shared correlations between DNA methylation and alternative splicing, and also a high abundance of tissue-specific effects.

In order to test whether DNA methylation is associated to alternative splicing, we performed Spearman rank correlation (SRC) between alternative splicing levels (also measured with *Altrans*, see Materials and Methods) and methylation levels of sites within 50 kb on either side of the TSS. Of the 5,124–6,020 tested genes, 4,602, 5,663 and 81 genes have significant associations between methylation and alternative splicing at 10% FDR (asQTM, alternative splicing Quantitative Trait Methylation) in fibroblasts, LCLs and T-cells, respectively (Table 1, S1 Dataset; see Materials and Methods). The substantially smaller number of significant associations found in T-cells may be due to sample size. Interestingly, similar to asQTLs, methylation sites associated to alternative splicing in LCLs and fibroblasts are significantly enriched for distinct regulatory regions such as active promoter and enhancer marks, transcription factor binding peak motifs, CTCF binding peaks and DNase I hypersensitive sites. They are also enriched for exons in general, but more strongly for middle exons, for elongation chromatin marks, and to a lesser extent to CpG islands (Fig. 6A; S6 Table). LCL and fibroblast eQTMs are as well significantly depleted in poised promoter marks and repressive marks (S6 Table). Enrichments for T-cell asQTMs were only significant for promoters, CTCF binding peaks, first exons and CpG island shores (Fig. 6A; S6 Table).

asQTM effects are considerably shared (π_1_: 0.46 to 0.75) in almost all comparisons (Table 2, S19B Fig.). They are not well shared when taking the significant associations in fibroblasts or LCLs and looking at the P-value distribution in T-cells (0.002 to 0.003; Table 2), reflecting the small number of discoveries possibly driven by small sample size. This lower abundance of associations in T-cells is also reflected in the comparisons of effect sizes among cell-types (Fig. 6B, S21 Fig.). However, when comparing effect sizes between fibroblasts and LCL associations, we get a picture comparable to asQTLs, where *R* ^2^ is 0.31 and 0.83 for union of and shared associations, respectively (Fig. 6B; S1 Table). These results reflect a significant amount of sharing among cell-types but an important amount of tissue-specificity too.

We conclude that genetic variation and DNA methylation are extensively associated to alternative splicing and these associations also present a wide spectrum in degree of tissue-specificity. Furthermore, enrichment of asQTLs and asQTMs in CTCF binding sites and exons may suggest a mechanism where methylation-sensitive CTCF binding affects alternative splicing [35]. Additionally, enrichment of asQTLs and asQTMs in both proximal and distant regulatory elements enforces studies that have shown that promoter architecture can influence alternative splicing (reviewed in [43]) and that exons often physically interact with promoters and enhancers, and these interactions correlate with alternative splicing events [44].

## Discussion

In this study we have provided high resolution analysis of tissue-specificity of allele specific expression and of the genetic and epigenetic (DNA methylation variation) associations to gene expression levels and alternative splicing, taking advantage of the large scale data for a population of individuals in three cell types. A large number of genetic variants affect gene expression levels, DNA methylation, and alternative splicing in a cell-type dependent manner. Additionally, we observe that although there is a significant tissue-specific effect on allele specific expression, the individual component is a higher determinant of allelic imbalance. This reflects the predominance of the genetic contribution to allele specific expression, over which epigenetic factors can then contribute to the tissue differences.

In line with this, we find that methylation can be associated to gene expression in a positive or negative manner that is highly replicated across cell-types, but the effect sizes of these associations appear more cell-type specific than genetic effects on expression and DNA methylation. The methylation levels that we have measured could involve not only methylation of cytosines but also hydroxymethylation, which is a mark that has recently been shown to be more common than anticipated, although on average in low levels [45, 46, 47]. Hence, future studies will need to further disentangle the positive and negative associations between DNA methylation and gene expression.

Finally, we show that DNA methylation is extensively associated to alternative splicing across the genome, and many of these associations present cell-type specificity. Our discoveries on genetic and methylation associations to alternative splicing highlight a scenario in which splicing can be dependent not only on factors occurring within the gene, but also on factors acting in both promoter proximal and distant regulatory regions. Given that DNA methylation, gene expression and alternative splicing changes are implicated in many diseases [13, 24, 48, 49, 50], characterizing the genetic causes of their inter-individual variation provides biological insights and mechanistic clues to the underlying pathophysiology of complex diseases and traits.

## Materials and Methods

Sample collection, cell growth, experiments (genotype, RNA-seq and DNA methylation assays), data processing and association tests performed for eQTLs, mQTLs and eQTMs are fully described in [37]. Here we summarize relevant information for these aspects and fully explain methods used for analyses fulfilled for this study.

### Ethics statement

Informed consent was obtained from all human subjects. The local ethics committee at University Hospitals of Geneva has approved this project (CER 10–046).

### Genotyping

204 GenCord individuals were SNP genotyped with the Illumina 2.5M Omni chip. After filtering, 1.5 million variants were imputed into the European panel SNPs of the 1000 genomes Phase 1 release [51] using Beagle v3.3.2 [52] yielding 6.9 million SNPs.

### RNA-sequencing and expression quantification

Total RNA was extracted from LCLs, fibroblasts and T-cells and mRNA-enriched cDNA libraries were sequenced with Illumina HiSeq2000 or Genome Analyzer II. 49bp paired-end reads were mapped to the genome with BWA [53]. Uniquely mapped, properly paired, MAPQ > = 10 reads mapping to merged exons from the GENCODE annotation v10 [54] were counted. Exons that were considered expressed are those that have at least one read mapped in at least 90% of the individuals studied. Raw exon counts were scaled to 10M reads per library and corrected for GC content, insert size mode, primer index and run date by linear regression. A median of 16 million reads per sample mapped to exons, which yielded sets of 70,800–76,870 normalized expressed exons, belonging to 12,265–12,863 genes.

### DNA methylation quantification

DNA was extracted from LCLs, fibroblasts and T-cells, bisulfite converted and processed through the 450K Illumina Infinium HD Methylation Assay according to manufacturer’s instructions. Probes with 1000 genomes project SNPs and indels at minor allele frequency >5% or any GenCord SNPs were filtered out. Data was quantile-normalized and the β-value was chosen as measure of fraction of DNA methylation per CpG site [55]. A final set of 416,117 CpG sites was used for analyses.

### Association analyses and multiple test correction

Spearman rank correlation was performed between all the pair wise combinations of genotypes, exon expression levels and DNA methylation levels using the window sizes indicated in Table 1. Analyses involving genotypes were done excluding genetic outlier individuals and including SNPs with minor allele frequency of >5%. Multiple testing correction was done by permuting expression or DNA methylation levels 1000 times of 1000 genes or 50000 CpG sites and extracting the median P-value distribution for determining significance (for each gene the minimum P-value distribution out of all its exons was used). A different process was followed for associations to alternative splicing (see below).

### Allele specific expression analyses

Allele-specific expression analysis was based on binomial testing of allelic ratios over heterozygous sites of each individual. Sites with at least 16 reads mapping with MAPQ > = 10 were tested, excluding those prone to mapping bias [5] (S13 Fig., S2 Table). Ratios were corrected for reference mapping bias and nucleotide-specific biases per individual library. The same analysis was repeated with the difference that we required at least 30 reads of coverage per site, and further sampling exactly 30 reads per site (Fig. 3A, Table 2).

We calculated allelic ratio distances between all sample pairs (individual—cell type) as the median of absolute REFERENCE/TOTAL ratio differences of all the shared heterozygous sites covered by > = 40 reads in both samples, weighted by the total number of reads covering the site (Fig. 3B). The higher coverage threshold was chosen to avoid random fluctuations of ratios due to low counts. This metric does not assume that the over-expressed allele is the same between samples. It intentionally captures also difference due to change of direction between individuals, as we think it is a valid type of biological variation, driven by difference linkage of regulatory and ASE variants. In these analyses, we excluded 12 samples where lower coverage led to the distances being based on fewer sites.

### Genomic feature enrichment analyses

The coordinates for the following genomic features were downloaded from the UCSC genome browser tables [56] and are part of ChIP-seq or DNAseI-seq experiments of the ENCODE project [57, 58, 59] and of particular groups, some of which have annotated regulatory elements by learning of chromatin states with ChromHMM [60, 61, 62, 63]: enhancers, insulators, elongation regions, poised promoters, active promoters, repressed regions, CTCF binding peaks and DNase I hypersensitive sites. We used data from the cell lines GM12878 and NHLF, for LCLs and fibroblasts, respectively. For T-cells, we used merged DNase I hypersensitive sites reported for Adult CD4+ Th0 and Th1. Given that there was no T-cell specific chromatin marks reported at the time of analysis, the data of GM12878, a closely related cell-type, were used instead.

Gene promoters go from −1kb to +2kb relative to the TSS. Gene bodies go from +2kb, relative to the TSS, to the end of the gene. CpG islands were downloaded from the UCSC genome browser [56]. CpG island shores are composed of the upstream and downstream 2kb regions flanking CpG islands. SNPs reported by Genome Wide Association Studies (GWAS) were downloaded from the NHGRI catalog (accessed on 30 April 2012)[41]. The splice region variants were taken from the Phase 1 1000 genomes variants annotation using the Variant Effector Predictor and Ensembl [51, 64, 65]. The transcription factor binding peak motifs are based on the ENCODE data and the file was taken from the Phase 1 1000 genomes annotation sets [51, 59]. We used BEDTools v2.7.1 [66] for processing many of the genomic features analyzed.

For eQTLs, mQTLs and asQTLs we assessed the enrichment of the best associated SNP per gene, CpG site or link, respectively, in distinct genomic features by comparing the overlaps observed in associated SNPs and null SNPs, and performing Fisher’s exact test to assess significance. The sets of null SNPs were chosen by requiring similar distance, minor allele frequency and expression or DNA methylation levels to that found in eQTL and mQTL SNPs, respectively. We further required that the null SNPs are not significantly (P < 0.01) associated to gene expression (for the null eQTL set) or DNA methylation (for the null mQTL set). For the set of null SNPs for the asQTLs we required similar distance and minor allele frequency to the asQTL SNPs.

For pos-eQTMs, neg-eQTMs, and asQTMs (top per link), we assessed the enrichment in distinct genomic features by comparing the overlaps observed in associated CpG sites and the null set of CpG sites, and performing Fisher’s exact test to assess significance. The null sets in this case are all non-eQTM or non-asQTM sites in the array that are within 50kb of a TSS.

### Discordant eQTM manual check

We found 12 eQTM associations with opposite direction between fibroblasts and T-cells. Only 2 of them are singletons, where one CpG site is associated to one exon. However, one of these exons codifies for alternate processed transcripts, and the other exon codifies for two different protein-coding genes. This raises the possibility that different transcripts are being quantified in each cell-type and the same methylation site would be associated to them in opposite ways. The other 10 associations are highly likely to be true positives. In one example, a CpG site is associated to 4 different exons of the same gene in the same consistent direction within each tissue (to note, all of these exons code for different isoforms). In another case two different CpG sites are associated to a single exon in the same consistent direction within each cell-type (this exon also codes for different isoforms). In a third case, two CpG sites are associated to the same two exons of a gene in a consistent manner within each tissue (i.e. four positive associations in fibroblasts and four negative associations in T-cells). These two exons of the same gene also code for different isoforms. In order to test whether these exons coding for several isoforms and presenting discordant associations were indeed being expressed in different transcripts between fibroblasts and T-cells, we used the *Altrans* method described below to test for differential exon-exon link usage. We were able to quantify links for 5 of these exons, and found highly significant differential link usage between cell-types for all of them. They all have *P*-values < 2.28E-67 (t-test) which is the median of 1000 randomly selected exons. Hence, they are more strongly differentiated than at least half of the other exons selected, however, there is a high level of tissue-specific isoforms between fibroblasts and T-cells in general.

### Alternative splicing quantification

We have developed a novel method for the relative quantification of splicing events that utilizes the paired-end nature of the RNA-seq experiment (Ongen H., Dermitzakis E.T., *submitted*). It uses the paired-end reads, where one read maps to one exon and the other read to a different exon, to count “links” between two exons. The first exon in a link is referred to as the “primary exon”. Overlapping exons are grouped into “exon groups” and unique portions of each exon in an exon group are identified, and subsequently used to assign reads to an exon. The raw link counts were normalized utilizing the effects of the first 15 principal components of these counts. The normalized link counts ascertained from unique regions of exons, which can be derived from parts of the linked exons rather than the whole exons, are divided by the probability of observing such a link given the empirically determined insert size distribution for each sample and unique portions of the exons in question, which is referred to as “link coverage”. Finally, the quantitative metric produced is the fraction of one link’s coverage over the sum of the coverages of all the links that the primary exon makes. We calculated this metric in 5’-to-3’ (forward) and 3’-to-5’ (reverse) directions to capture splice acceptor and donor effects respectively. In the association analyses, we only included links where the primary exon’s exon group made at least 10 links in the analyzed direction in at least 80% of the individuals and where the primary exon made at least 5 links in the analyzed direction in at least 30% of the individuals. Furthermore, links with more than 95% non-variable values across individuals were filtered out. For calling asQTLs, we have permuted the link ratios and then correlated these to the genotypes in order to calculate an empirical P-value. Specifically, we have permuted the link ratios 100 to a maximum of 100,000 times until we obtain 100 times a permutation P-value lower than the observed nominal P-value. We then calculate the empirical P-value by dividing the number of permutation P-values below the nominal P-value divided by the number of permutations performed. Subsequently, we used the qvalue R package on these empirical P-values for correcting for multiple testing [38].

## Supporting Information

S1 Table Summary of effect size variability analyses. (DOCX)

S2 Table π_1_ statistic, representing fraction of effects shared between cell-types, for sites with at least 16 reads. (DOCX)

S3 Table Assessment of enrichment of eQTMs in distinct genomic regions. (DOCX)

S4 Table eQTMs significant in both fibroblasts and T-cells, and with opposite sign of correlation (discordant). (DOCX)

S5 Table Assessment of enrichment of asQTLs in distinct genomic regions. (DOCX)

S6 Table Assessment of enrichment of asQTMs in distinct genomic regions. (DOCX)

S1 Dataset Discovered asQTLs and asQTMs at 10% FDR. (ZIP)

S1 Fig Replication of Gencord reported LCL eQTLs in another study. We have estimated how our LCL eQTL results replicate in an independent dataset recently published (the Geuvadis dataset, including 373 European individuals, by Lappalainen *et al*., 2013). (A) We have taken Gencord eQTLs and plotted the P-value distribution in Geuvadis, yielding a π_1_ of 0.69 (estimated fraction of true positives). (B) Plotted are the effect sizes for the Gencord eQTLs in Gencord (x-axis) and in Geuvadis (y-axis). The Spearman rank correlation coefficient between effect sizes is 0.73.
(TIF)

S2 Fig Clustering of eQTLs around the TSS, by approximation to degree of tissue specificity. (A) Relative distance of SNP to the transcription start site (TSS; x-axis) by—log(P-value) (y-axis) of (from left to right) all eQTLs (best per gene), eQTLs genes discovered (significant) in one cell-type, eQTL genes discovered in two cell-types, eQTLs genes discovered in all three cell-types, in (from top to bottom) fibroblasts (F), LCLs (L), and T-cells (T). (B) Distributions of absolute eQTL distance to TSS for eQTLs called significant in one, two or three cell-types. (C) Distributions of absolute eQTL distance to TSS for LCL replicated eQTLs (P < 0.05 in Geuvadis) that are discovered in only LCLs (1), in LCLs and another cell-type (2), in LCLs and the two other cell-types (3). (D) Union of SNP-exon eQTLs of fibroblasts, LCLs and T-cells (whose SNP and exon were tested in all three cell-types) that have a P<0.05 in one, two or three cell-types.
(TIF)

S3 Fig Distribution of eQTLs before the TSS, in the gene and at the TES. (A) Plotted are the distributions of eQTLs upstream of the transcription start site (TSS), inside the gene and downstream of the transcription end site (TES) by assessing the proportion of QTLs falling in 10kb windows for upstream of TSS or downstream of TES, and deciles inside the gene (i.e. all genes fitted to one size). We observe a high proportion of eQTLs not only close to the TSS, but also at the TES. (B) Using the same bins as described for (A) (x-axis), we have plotted the fold enrichment for cell-type specific eQTLs (genes called significant in only one cell-type; positive y-axis) and the fold enrichment for shared eQTLs (called significant in at least 2 cell-types; negative y-axis; i.e. a value of −2 is a 2 fold enrichment for shared eQTLs). This pattern observed further supports the notion that eQTLs away from the gene tend to be more cell-type specific, whereas the gene body and proximal flanking regions are enriched for shared eQTLs. However, this classification method for approximation to cell-type specificity presents some caveats (see main text).
(TIF)

S4 Fig Distance of mQTLs to CpG site by level of significance. Relative distance of SNP to the CpG site (x-axis) by—log(P-value) (y-axis) in fibroblasts (F), LCLs (L) and T-cells (T; left panels). Distribution of distance to CpG site (y-axis) in expected set (random uniform), observed mQTLs at 10% FDR and highly significant mQTLs (top 25%) in each cell-type (right panels). All differences between distributions are significant, with all P < 1.3E-14 (Wilcoxon’s test).
(TIF)

S5 Fig Assessment of enrichment of eQTLs in distinct genomic regions. For distinct genomic regions, the proportion of overlapping eQTLs was compared to the proportion of overlapping null SNPs. One star indicates *P* < 0.05, two stars indicate *P* < 5E-04, Fisher’s exact test. Fibroblasts (F), LCLs (L), T cell (T).
(TIF)

S6 Fig Assessment of enrichment of mQTLs in distinct genomic regions. For distinct genomic regions, the proportion of overlapping mQTLs was compared to the proportion of overlapping null SNPs. One star indicates *P* < 0.05, two stars indicate *P* < 5E-04, Fisher’s exact test. Fibroblasts (F), LCLs (L), T cell (T). To note, although there is a significant depletion of mQTLs in promoters in all three cell-types, this has been shown to be different depending on whether the promoter has a CpG island or not [37].
(TIF)

S7 Fig Overlap of eQTLs and mQTLs across cell-types. Overlap of eQTLs (A) and mQTLs (B) illustrated by the relative amount of eQTL genes or mQTL sites in each cell-type (x-axis) found significant in one, two or three cell-types (y-axis). Number of eQTL genes or mQTL sites falling in each category is indicated in the squares.
(TIF)

S8 Fig Direction of effect comparisons between cell-types for eQTLs and mQTLs. Correlation coefficients of eQTLs (A) or mQTLs (B) significant in both cell-types compared for all pair wise combinations of the three cell-types. Percentage of discordant cases (associations with opposite direction between any pair of cell-types) is indicated in the top left corner of each panel.
(TIF)

S9 Fig eQTL effect size calculation example. Distribution of expression levels of an exon (top) and its eQTL (bottom) for normal (A) and scaled (B) data. Scaled data is calculated by subtracting the mean and dividing by the standard deviation of normal data. Effect size of eQTLs is the difference between the median scaled expression level of the homozygous individuals for the major allele and the median scaled expression level of the heterozygous individuals (medians depicted by red lines). The effect size of this example is 0.56.
(TIF)

S10 Fig mQTL effect size calculation example. Distribution of DNA methylation levels of a CpG site (top) and its mQTL (bottom) for normal (A) and scaled (B) data. Scaled data is calculated by subtracting the mean and dividing by the standard deviation of normal data. Effect size of mQTLs is the difference between the median scaled methylation level of the homozygous individuals for the major allele and the median scaled methylation level of the heterozygous (medians depicted by red lines). The effect size of this example is −0.9.
(TIF)

S11 Fig eQTL and mQTL effect size distributions. Histograms depicting the effect sizes distributions of eQTLs (left panels) and mQTLs (right panels) in each cell-type.
(TIF)

S12 Fig eQTL and mQTL effect sizes when significant in one or multiple cell-types. Absolute effect size distributions (y-axis) of eQTLs (A) and mQTLs (B) when the gene or CpG site has a significant eQTL or mQTL, respectively, in one, two or three cell-types (x-axis), in fibroblasts (F), LCLs (L) and T-cells (T).
(TIF)

S13 Fig Allele specific expression summary statistics and overlapping among tissues using 16 read cut-off. (A-E) Results of ASE analyses requiring at least 16 reads per site. (A) Distribution of number of assayed heterozygous sites per cell-type. (B) Distribution of proportion of both alleles seen (BAS) per site in each cell-type. (C) Distribution of number of allele specific expressed (ASE) sites (P < 0.005) per cell-type. (D) Distribution of percent of heterozygous sites assayed with significant ASE per sample, in each cell-type. (E) Sharing of Allele-Specific Expression (ASE) illustrated by the relative amount of ASE sites (P < 0.005) in each cell-type (x-axis) found significant in one, two or three cell-types within each individual (y-axis). Number of ASE sites falling in each category is indicated in the squares. F, L and T stand for fibroblasts, LCLs and T-cells, respectively.
(TIF)

S14 Fig eQTM gene overlap across cell-types. Overlap of eQTMs illustrated by the relative amount of eQTM genes in each cell-type (x-axis) found in one, two or three cell-types (y-axis). Number of eQTM genes falling in each category is indicated in the squares. 44–63% of genes with eQTMs are observed in at least two cell-types.
(TIF)

S15 Fig eQTM effect size calculation examples. Two examples of a pos-eQTM (A) and a neg-eQTM (B) showing how effect sizes are calculated. In each (A) and (B), the top histograms show the distributions of expression level of an exon and DNA methylation levels of a CpG site that are significantly associated (top scatterplot). The bottom histograms show the same distributions for scaled data. Scaled data is calculated by subtracting the mean and dividing by the standard deviation. Effect size of eQTMs is calculated as the linear regression slope of scaled data of expression given methylation, as depicted in the bottom scatterplots on the right with the slope depicted in red and the effect size indicated above the plot.
(TIF)

S16 Fig eQTM effect size distributions. Histograms depicting the distribution of eQTM effect sizes in each cell-type.
(TIF)

S17 Fig Replication of Gencord reported LCL asQTLs in another study. We have estimated how our LCL asQTL results replicate in an independent dataset recently published (the Geuvadis dataset, including 373 European individuals, by Lappalainen *et al*., 2013). (A) We have taken Gencord asQTLs and plotted the P-value distribution in Geuvadis, yielding a π_1_ of 0.66 (estimated fraction of true positives). (B) Plotted are the effect sizes for the Gencord asQTLs in Gencord (x-axis) and in Geuvadis (y-axis). The Spearman rank correlation coefficient between effect sizes is 0.6.
(TIF)

S18 Fig Distribution of asQTLs before the TSS, in the gene and after the TES. Plotted are the distributions of asQTLs (best per exon-exon link) upstream of the transcription start site (TSS), inside the gene and downstream of the transcription end site (TES) by assessing the proportion of asQTLs falling in 10kb windows for upstream of TSS or downstream of TES, and deciles inside the gene (i.e. all genes fitted to one size). We observe a high proportion of asQTLs close to the TSS and also some enrichment at the TES.
(TIF)

S19 Fig Overlap of DNA methylation and genetic variation associations to alternative splicing among cell-types. Overlap of asQTLs (A) and asQTMs (B) illustrated by the relative amount of asQTL genes or asQTM genes in each cell-type (x-axis) found significant in one, two or three cell-types (y-axis). Numbers falling in each category are indicated inside the boxes.
(TIF)

S20 Fig asQTL effect size distributions and examples. (A) Distributions of asQTL effect sizes in fibroblasts (F), LCLs (L) and T-cells (T) (left panels), with a close up vision on the center of the distributions (right panels). Effect sizes are calculated as the difference in medians of scaled alternative splicing levels between heterozygous individuals and individuals homozygous for the major allele. Scaled data is calculated by subtracting the mean and dividing by the standard deviation of normal data. (B) Plotted are examples of asQTLs with one shown for each cell-type, with the effect size indicated above each plot.
(TIF)

S21 Fig asQTM effect size distributions and examples. (A) Distributions of asQTM effect sizes in fibroblasts, LCLs and T-cells. (B) Plotted are examples of asQTMs in each cell-type, with the effect size indicated on the top of each plot. Effect size of asQTMs is calculated as the linear regression slope of scaled data of alternative splicing levels given DNA methylation levels, as depicted in red in the scatter plots. Scaled data is calculated by subtracting the mean and dividing by the standard deviation of normal data.
(TIF)
