# Supplementary material for: Tissue-Specific Effects of Genetic and Epigenetic Variation on Gene Regulation and Splicing
Source: PLoS Genet. 2015 Jan 29;11(1):e1004958. doi: 10.1371/journal.pgen.1004958 (PMC4310612; doi:10.1371/journal.pgen.1004958)
Supplement: S1 Table — (DOCX) [file pgen.1004958.s001.docx]

*Table S1: Summary of effect size variability analyses*

|  | **Cell-types compared** | **Total in Union** | **R2 of Union** | **Total Significant in Both** | **Percent Significant in Both** | **R2 of Significant in Both** |
| --- | --- | --- | --- | --- | --- | --- |
| **eQTLs**  (best per gene, SNP-exon pairs) | Fibroblasts-LCLs | 4623 | 0.23 | 83 | 1.8 | 0.9 |
|  | Fibroblasts-T-cells | 3576 | 0.2 | 75 | 2.1 | 0.92 |
|  | LCLs-T-cells | 4749 | 0.37 | 142 | 2.99 | 0.88 |
| **mQTLs**  (best per CpG site, SNP-CpG pairs) | Fibroblasts-LCLs | 34092 | 0.22 | 2379 | 6.98 | 0.79 |
|  | Fibroblasts-T-cells | 43510 | 0.18 | 2559 | 5.88 | 0.7 |
|  | LCLs-T-cells | 46914 | 0.47 | 7196 | 15.34 | 0.78 |
| **eQTMs**  (all significant per gene, CpG-exon pairs) | Fibroblasts-LCLs | 12321 | 0.009 | 111 | 0.90 | 0.866 |
|  | Fibroblasts-T-cells | 13723 | 0.003 | 87 | 0.63 | 0.547 |
|  | LCLs-T-cells | 25839 | 0.024 | 700 | 2.71 | 0.666 |
| **asQTLs**  (best per exon-exon link, SNP-link pairs) | Fibroblasts-LCLs | 971 | 0.342 | 34 | 3.50 | 0.83 |
|  | Fibroblasts-T-cells | 995 | 0.455 | 32 | 3.22 | 0.701 |
|  | LCLs-T-cells | 1652 | 0.424 | 96 | 5.81 | 0.894 |
| **asQTMs**  (best per exon-exon link, CpG-link pairs) | Fibroblasts-LCLs | 129776 | 0.307 | 4489 | 3.46 | 0.832 |
|  | Fibroblasts-T-cells | 52539 | 4.19E-07 | 4 | 0.01 | 0.147 |
|  | LCLs-T-cells | 94076 | 3.51E-06 | 12 | 0.01 | 0.933 |
