# Supplementary material for: Tissue-Specific Effects of Genetic and Epigenetic Variation on Gene Regulation and Splicing
Source: PLoS Genet. 2015 Jan 29;11(1):e1004958. doi: 10.1371/journal.pgen.1004958 (PMC4310612; doi:10.1371/journal.pgen.1004958)
Supplement: S2 Table — (DOCX) [file pgen.1004958.s002.docx]

*Table S2: π1 statistic, representing fraction of effects shared between cell-types, for sites with at least 16 reads.*

|  | significant in – π1 of P-value distribution in | | | | | |
| --- | --- | --- | --- | --- | --- | --- |
|  | **F-L** | **F-T** | **L-F** | **L-T** | **T-F** | **T-L** |
| **>=16 reads** | 0.26 | 0.34 | 0.36 | 0.43 | 0.38 | 0.46 |
