# Supplementary material for: Tissue-Specific Effects of Genetic and Epigenetic Variation on Gene Regulation and Splicing
Source: PLoS Genet. 2015 Jan 29;11(1):e1004958. doi: 10.1371/journal.pgen.1004958 (PMC4310612; doi:10.1371/journal.pgen.1004958)
Supplement: S3 Table — (DOCX) [file pgen.1004958.s003.docx]

*Table S3: Assessment of enrichment of eQTMs in distinct genomic regions*

|  | **Fribroblasts** | | | | **LCLs** | | | | **T-cells** | | | |
| --- | --- | --- | --- | --- | --- | --- | --- | --- | --- | --- | --- | --- |
|  | **neg-eQTMs** | | **pos-eQTM** | | **neg-eQTMs** | | **pos-eQTMs** | | **neg-eQTMs** | | **pos-eQTMs** | |
|  | **Enrich** | **P-value** | **Enrich** | **P-value** | **Enrich** | **P-value** | **Enrich** | **P-value** | **Enrich** | **P-value** | **Enrich** | **P-value** |
| **TF motifs** | 0.9 | 0.461 | 0.6 | 0.007 | 0.9 | 0.089 | 0.8 | 0.00018 | 0.8 | 0.00029 | 0.9 | 0.111 |
| **CpG island shores** | 1.7 | 3.91E-18 | 1.3 | 3.21E-05 | 1.2 | 1.57E-24 | 1.0 | 0.257 | 1.3 | 1.40E-26 | 1.3 | 1.90E-19 |
| **CpG islands** | 0.9 | 0.148 | 1.0 | 0.512 | 0.9 | 1.10E-05 | 0.7 | 5.33E-47 | 0.6 | 1.25E-88 | 0.7 | 3.12E-39 |
| **All exons** | 1.2 | 0.014 | 1.2 | 0.010 | 1.4 | 4.58E-51 | 1.1 | 0.001 | 1.1 | 1.20E-06 | 1.1 | 1.72E-05 |
| **First exons** | 1.1 | 0.403 | 1.2 | 0.224 | 1.1 | 0.026 | 0.9 | 0.252 | 1.0 | 0.681 | 0.9 | 0.377 |
| **Last exons** | 0.9 | 0.656 | 1.1 | 0.729 | 1.1 | 0.088 | 1.0 | 0.792 | 1.0 | 0.397 | 0.8 | 0.013 |
| **Middle exons** | 1.3 | 0.003 | 1.2 | 0.020 | 1.5 | 1.43E-63 | 1.2 | 7.47E-07 | 1.2 | 5.50E-10 | 1.3 | 3.68E-13 |
| **Gene bodies** | 1.2 | 8.16E-06 | 1.5 | 1.44E-18 | 1.3 | 2.81E-60 | 1.4 | 1.76E-88 | 1.2 | 1.14E-18 | 1.3 | 2.16E-33 |
| **Promoters** | 1.0 | 0.711 | 0.7 | 1.05E-11 | 1.0 | 0.245 | 0.7 | 4.05E-61 | 1.0 | 0.121 | 0.9 | 1.48E-09 |
| **Introns** | 1.1 | 0.108 | 1.1 | 0.039 | 1.1 | 0.001 | 1.2 | 7.75E-15 | 1.1 | 1.47E-11 | 1.1 | 0.002 |
| **CTCF binding peaks** | 1.3 | 7.87E-05 | 0.9 | 0.094 | 1.1 | 2.64E-08 | 0.9 | 1.90E-05 | 1.2 | 8.49E-09 | 1.1 | 0.040 |
| **Active promoters** | 1.2 | 0.008 | 0.6 | 9.85E-08 | 1.1 | 7.69E-09 | 0.7 | 4.34E-21 | 0.9 | 0.044 | 0.8 | 6.09E-09 |
| **Elongation** | 0.9 | 0.394 | 1.9 | 8.39E-08 | 2.2 | 4.26E-69 | 0.6 | 3.41E-08 | 1.0 | 0.731 | 2.0 | 1.12E-24 |
| **Enhancers** | 2.1 | 1.62E-15 | 0.7 | 0.006 | 2.1 | 1.02E-129 | 1.2 | 7.46E-05 | 2.9 | 2.97E-274 | 2.1 | 2.32E-58 |
| **Insulators** | 0.9 | 1 | 0.6 | 0.209 | 0.6 | 2.06E-05 | 1.1 | 0.270 | 0.8 | 0.076 | 0.8 | 0.179 |
| **Poised promoters** | 0.5 | 0.034 | 0.6 | 0.111 | 0.4 | 2.29E-24 | 0.3 | 2.86E-24 | 0.4 | 2.73E-22 | 0.4 | 2.53E-15 |
| **Repressed** | 0.6 | 0.001 | 1.8 | 5.25E-10 | 0.2 | 1.36E-117 | 0.7 | 6.53E-13 | 0.5 | 1.15E-45 | 0.6 | 9.36E-17 |
| **Dnase I HSs** | 1.1 | 0.066 | 0.7 | 3.41E-06 | 1.1 | 1.20E-08 | 0.8 | 2.19E-21 | 1.1 | 2.06E-06 | 1.0 | 0.093 |
| **non-genic Dnase I HSs** | 1.0 | 0.830 | 0.6 | 0.002 | 0.9 | 0.003 | 0.7 | 2.49E-15 | 0.9 | 1.71E-05 | 0.8 | 4.41E-05 |
