# Supplementary material for: Tissue-Specific Effects of Genetic and Epigenetic Variation on Gene Regulation and Splicing
Source: PLoS Genet. 2015 Jan 29;11(1):e1004958. doi: 10.1371/journal.pgen.1004958 (PMC4310612; doi:10.1371/journal.pgen.1004958)
Supplement: S4 Table — (DOCX) [file pgen.1004958.s004.docx]

*Table S4: eQTMs significant in both fibroblasts and T-cells and with opposite sign of correlation (discordant).*

| Methylation Probe ID | Exon ID | Rho in F | Rho in T |
| --- | --- | --- | --- |
| cg08803663 | ENSG00000173295.2_8101621_8102384 | -0.502 | 0.527 |
| cg09365002 | ENSG00000204209.6_33286774_33286996 | -0.405 | 0.447 |
| cg09365002 | ENSG00000204209.6_33287157_33287631 | -0.428 | 0.431 |
| cg10141261 | ENSG00000182022.12_125767184_125769855 | 0.373 | -0.563 |
| cg15378605 | ENSG00000182022.12_125767184_125769855 | 0.428 | -0.503 |
| cg22822867 | ENSG00000178209.10_144989321_144996563 | 0.41 | -0.458 |
| cg22822867 | ENSG00000178209.10_144996672_145000052 | 0.371 | -0.446 |
| cg22822867 | ENSG00000178209.10_145000952_145001050 | 0.371 | -0.608 |
| cg22822867 | ENSG00000178209.10_145009366_145009481 | 0.376 | -0.436 |
| cg26500914 | ENSG00000204209.6_33286774_33286996 | -0.425 | 0.494 |
| cg26500914 | ENSG00000204209.6_33287157_33287631 | -0.421 | 0.46 |
| cg27485921 | ENSG00000119729.6_46808067_46810260 | 0.396 | -0.631 |
