# Supplementary material for: Tissue-Specific Effects of Genetic and Epigenetic Variation on Gene Regulation and Splicing
Source: PLoS Genet. 2015 Jan 29;11(1):e1004958. doi: 10.1371/journal.pgen.1004958 (PMC4310612; doi:10.1371/journal.pgen.1004958)
Supplement: S5 Table — (DOCX) [file pgen.1004958.s005.docx]

*Table S5: Assessment of enrichment of asQTLs in distinct genomic regions*

|  | **Fibroblasts** |  | **LCLs** |  | **T-cells** |  |
| --- | --- | --- | --- | --- | --- | --- |
|  | **Enrichment** | **P-value** | **Enrichment** | **P-value** | **Enrichment** | **P-value** |
| **non-genic DHSs** | 2.8 | 3.45E-05 | 3.6 | 5.72E-11 | 3.2 | 2.10E-06 |
| **GWAS SNPs** | 4.4 | 0.026 | 1.9 | 0.284 | 3.2 | 0.095 |
| **Active Promoters** | 2.5 | 7.72E-07 | 3.1 | 2.36E-17 | 3.5 | 1.96E-11 |
| **Enhancers** | 2.3 | 1.40E-11 | 3.0 | 6.49E-36 | 3.2 | 4.33E-28 |
| **DHSs** | 2.4 | 3.47E-08 | 2.7 | 1.52E-20 | 2.9 | 5.11E-21 |
| **TF motifs** | 2.3 | 0.056 | 2.8 | 7.30E-04 | 3.0 | 0.011 |
| **Middle exons** | 2.4 | 1.92E-06 | 2.5 | 9.11E-11 | 2.9 | 6.32E-11 |
| **CGIs** | 2.4 | 3.40E-05 | 2.2 | 2.78E-06 | 2.8 | 1.76E-07 |
| **All exons** | 2.1 | 6.56E-07 | 2.2 | 2.37E-12 | 2.4 | 3.42E-10 |
| **Last exons** | 1.9 | 0.049 | 2.3 | 3.97E-04 | 2.3 | 0.006 |
| **Elongation** | 2.6 | 4.00E-18 | 2.0 | 3.16E-15 | 1.5 | 7.23E-04 |
| **Insulators** | 2.2 | 0.004 | 1.7 | 0.141 | 2.2 | 0.040 |
| **CGI shores** | 1.9 | 6.50E-09 | 1.9 | 9.17E-12 | 2.0 | 4.65E-11 |
| **CTCF binding peaks** | 2.1 | 5.56E-11 | 1.7 | 8.17E-08 | 1.7 | 1.94E-05 |
| **First exons** | 1.2 | 0.605 | 1.5 | 0.067 | 2.0 | 0.019 |
| **Poised promoters** | 1.6 | 0.436 | 0.4 | 0.438 | 1.8 | 0.414 |
| **Promoters** | 1.1 | 0.590 | 1.4 | 1.69E-04 | 1.4 | 0.002 |
| **Introns** | 1.0 | 0.326 | 0.9 | 0.048 | 0.9 | 0.040 |
| **Repressed** | 1.4 | 0.005 | 0.6 | 0.003 | 0.5 | 0.003 |
| **Splice region variant** | 6.5 | 1.64E-05 | 14.5 | 3.18E-20 | 14.6 | 1.45E-12 |
