# Supplementary material for: Tissue-Specific Effects of Genetic and Epigenetic Variation on Gene Regulation and Splicing
Source: PLoS Genet. 2015 Jan 29;11(1):e1004958. doi: 10.1371/journal.pgen.1004958 (PMC4310612; doi:10.1371/journal.pgen.1004958)
Supplement: S6 Table — (DOCX) [file pgen.1004958.s006.docx]

*Table S6: Assessment of enrichment of asQTMs in distinct genomic regions*

|  | **Fibroblasts** |  | **LCLs** |  | **T-cells** |  |
| --- | --- | --- | --- | --- | --- | --- |
|  | **Enrichment** | **P-value** | **Enrichment** | **P-value** | **Enrichment** | **P-value** |
| **Active promoters** | 1.9 | 0.00E+00 | 1.9 | 0.00E+00 | 1.4 | 0.013 |
| **Elongation marks** | 1.3 | 0.000 | 2.2 | 0.000 | 1.1 | 0.681 |
| **CTCF binding peaks** | 1.4 | 2.69E-228 | 1.4 | 0.00E+00 | 1.5 | 0.003 |
| **Firs exons** | 1.1 | 4.80E-10 | 1.2 | 3.47E-20 | 1.9 | 7.64E-03 |
| **Middle exons** | 1.4 | 5.30E-148 | 1.5 | 2.30E-302 | 1.2 | 0.334 |
| **DHSs** | 1.6 | 0.00E+00 | 1.3 | 0.00E+00 | 1.2 | 0.103 |
| **TF motifs** | 1.6 | 4.80E-105 | 1.4 | 1.69E-63 | 1.0 | 1 |
| **Enhancer marks** | 1.3 | 9.45E-44 | 1.3 | 7.56E-56 | 1.3 | 0.181 |
| **DHSs non-genic** | 1.3 | 0.000 | 1.2 | 0.000 | 1.4 | 0.068 |
| **Promoters** | 1.3 | 0.00E+00 | 1.2 | 9.31E-155 | 1.3 | 3.51E-03 |
| **All exons** | 1.3 | 7.95E-127 | 1.3 | 3.95E-234 | 1.1 | 0.539 |
| **Last exons** | 1.2 | 8.77E-23 | 1.2 | 4.04E-28 | 1.0 | 0.861 |
| **CGIs** | 1.3 | 3.58E-250 | 1.1 | 5.17E-75 | 1.0 | 0.925 |
| **CGI shores** | 1.0 | 3.12E-01 | 1.0 | 7.79E-01 | 1.4 | 0.022 |
| **Introns** | 1.0 | 7.29E-01 | 1.0 | 2.67E-02 | 1.0 | 0.781 |
| **Insulator marks** | 0.7 | 5.68E-11 | 0.7 | 3.36E-16 | 0.5 | 1 |
| **Poised promoter marks** | 0.4 | 6.57E-117 | 0.3 | 4.13E-248 | 0.7 | 0.665 |
| **Repressed marks** | 0.4 | 0.00E+00 | 0.2 | 0.000 | 0.7 | 0.386 |
