# Supplementary figures and images for: Tissue-Specific Effects of Genetic and Epigenetic Variation on Gene Regulation and Splicing
Source: PLoS Genet. 2015 Jan 29;11(1):e1004958. doi: 10.1371/journal.pgen.1004958 (PMC4310612; doi:10.1371/journal.pgen.1004958)

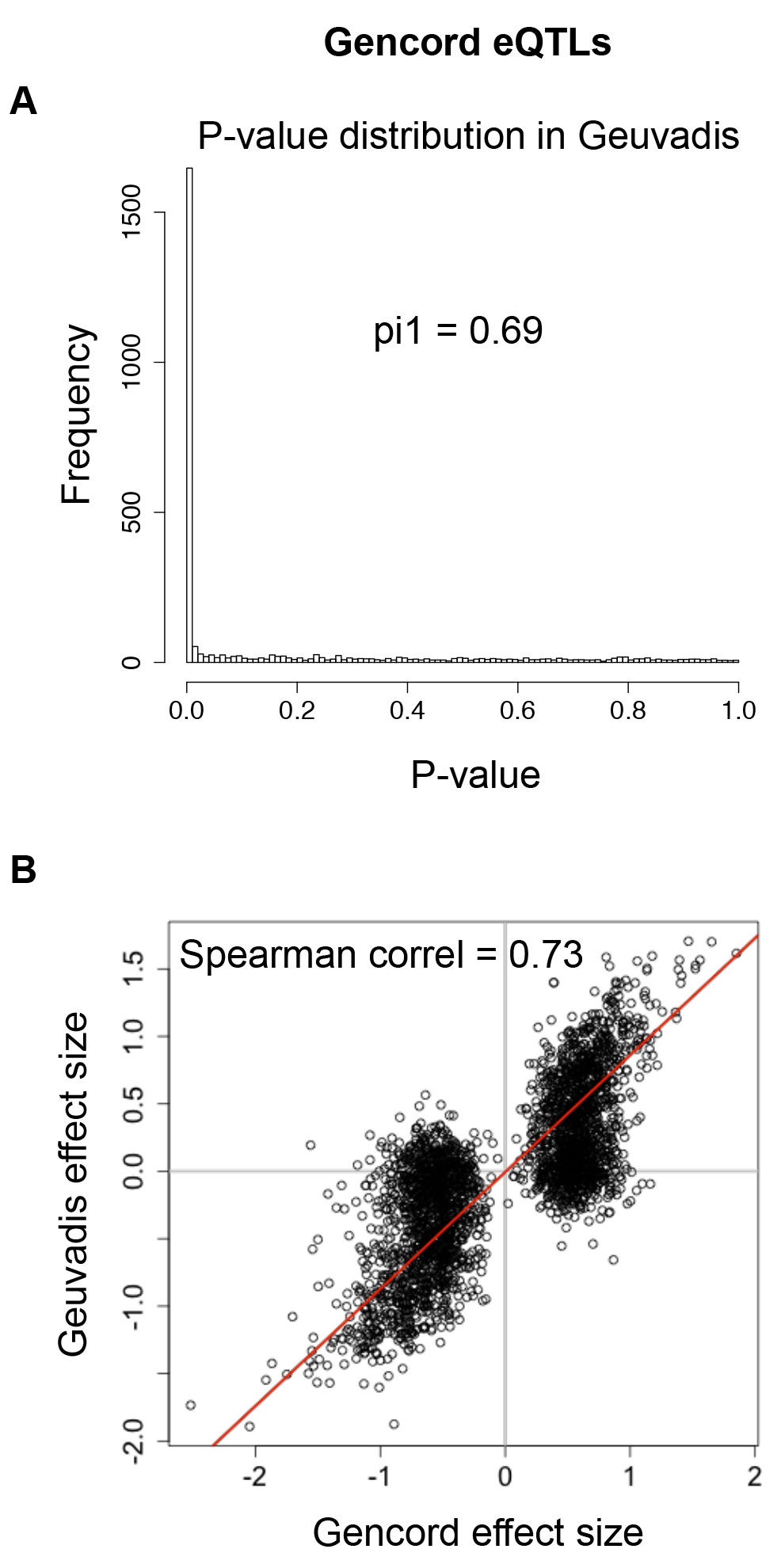

Supplement: S1 Fig — We have estimated how our LCL eQTL results replicate in an independent dataset recently published (the Geuvadis dataset, including 373 European individuals, by Lappalainen et al., 2013). (A) We have taken Gencord eQTLs and plotted the P-value distribution in Geuvadis, yielding a π1 of 0.69 (estimated fraction of true positives). (B) Plotted are the effect sizes for the Gencord eQTLs in Gencord (x-axis) and in Geuvadis (y-axis). The Spearman rank correlation coefficient between effect sizes is 0.73. (TIF) [file pgen.1004958.s008.tif]

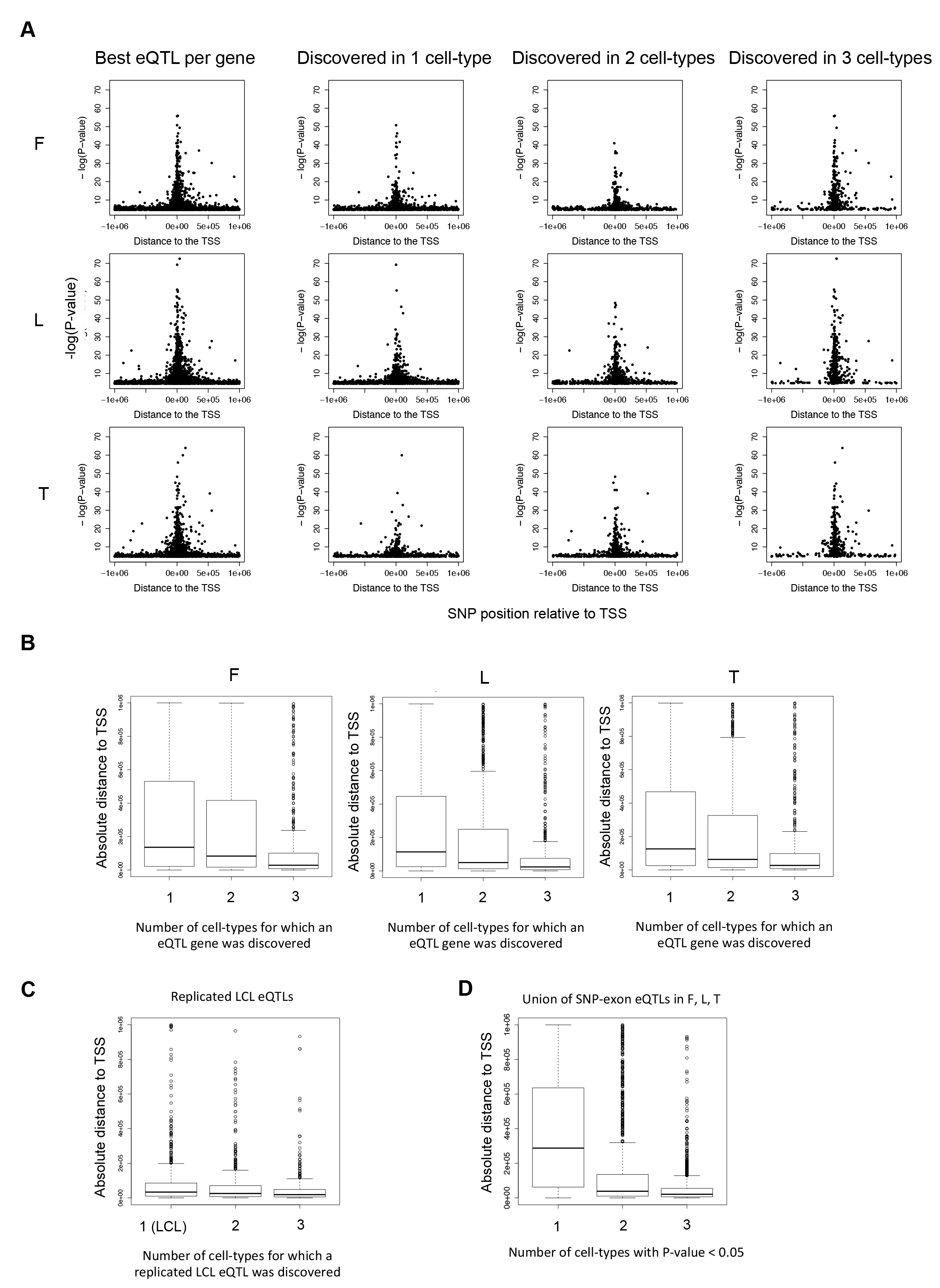

Supplement: S2 Fig — (A) Relative distance of SNP to the transcription start site (TSS; x-axis) by—log(P-value) (y-axis) of (from left to right) all eQTLs (best per gene), eQTLs genes discovered (significant) in one cell-type, eQTL genes discovered in two cell-types, eQTLs genes discovered in all three cell-types, in (from top to bottom) fibroblasts (F), LCLs (L), and T-cells (T). (B) Distributions of absolute eQTL distance to TSS for eQTLs called significant in one, two or three cell-types. (C) Distributions of absolute eQTL distance to TSS for LCL replicated eQTLs (P < 0.05 in Geuvadis) that are discovered in only LCLs (1), in LCLs and another cell-type (2), in LCLs and the two other cell-types (3). (D) Union of SNP-exon eQTLs of fibroblasts, LCLs and T-cells (whose SNP and exon were tested in all three cell-types) that have a P<0.05 in one, two or three cell-types. (TIF) [file pgen.1004958.s009.tif]

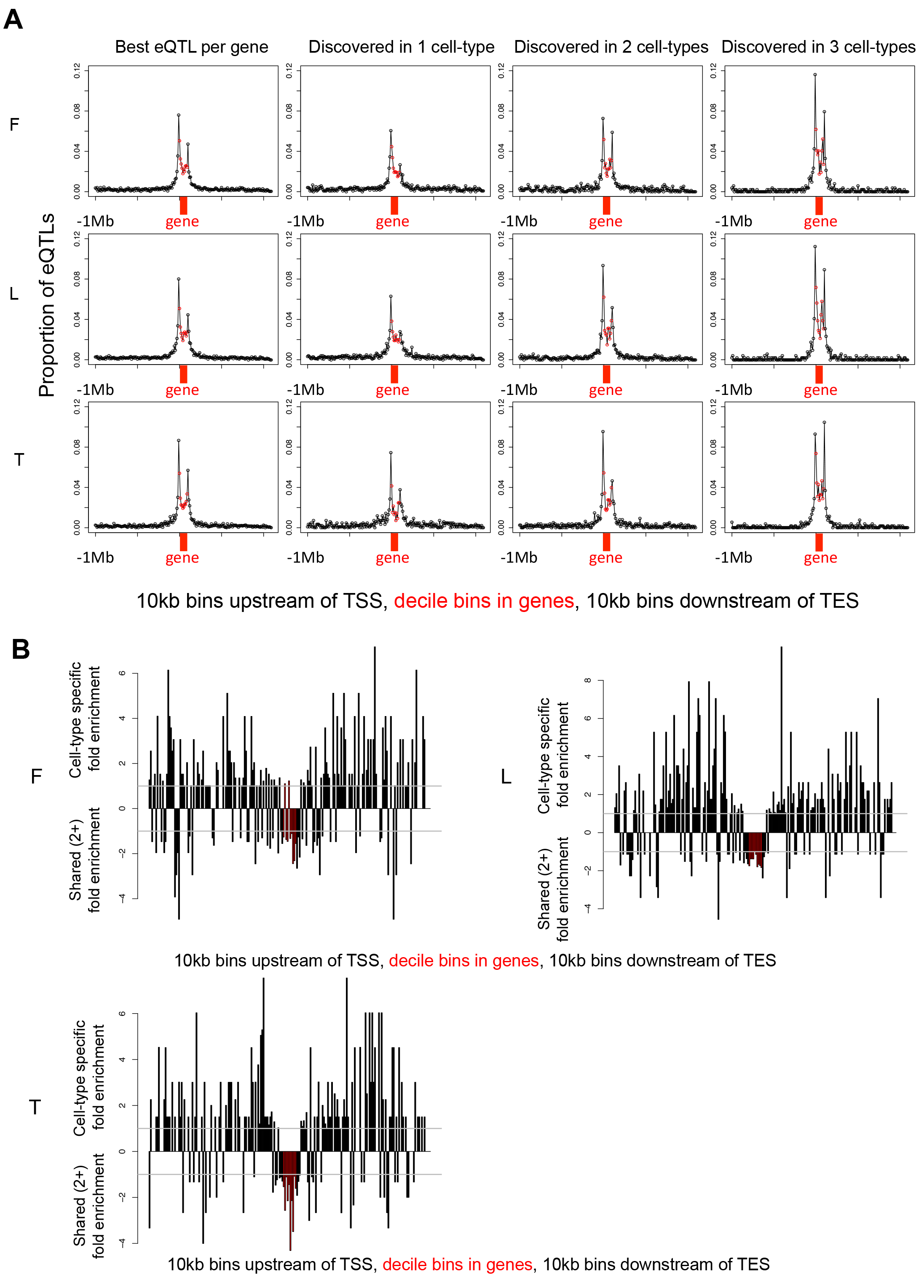

Supplement: S3 Fig — (A) Plotted are the distributions of eQTLs upstream of the transcription start site (TSS), inside the gene and downstream of the transcription end site (TES) by assessing the proportion of QTLs falling in 10kb windows for upstream of TSS or downstream of TES, and deciles inside the gene (i.e. all genes fitted to one size). We observe a high proportion of eQTLs not only close to the TSS, but also at the TES. (B) Using the same bins as described for (A) (x-axis), we have plotted the fold enrichment for cell-type specific eQTLs (genes called significant in only one cell-type; positive y-axis) and the fold enrichment for shared eQTLs (called significant in at least 2 cell-types; negative y-axis; i.e. a value of −2 is a 2 fold enrichment for shared eQTLs). This pattern observed further supports the notion that eQTLs away from the gene tend to be more cell-type specific, whereas the gene body and proximal flanking regions are enriched for shared eQTLs. However, this classification method for approximation to cell-type specificity presents some caveats (see main text). (TIF) [file pgen.1004958.s010.tif]

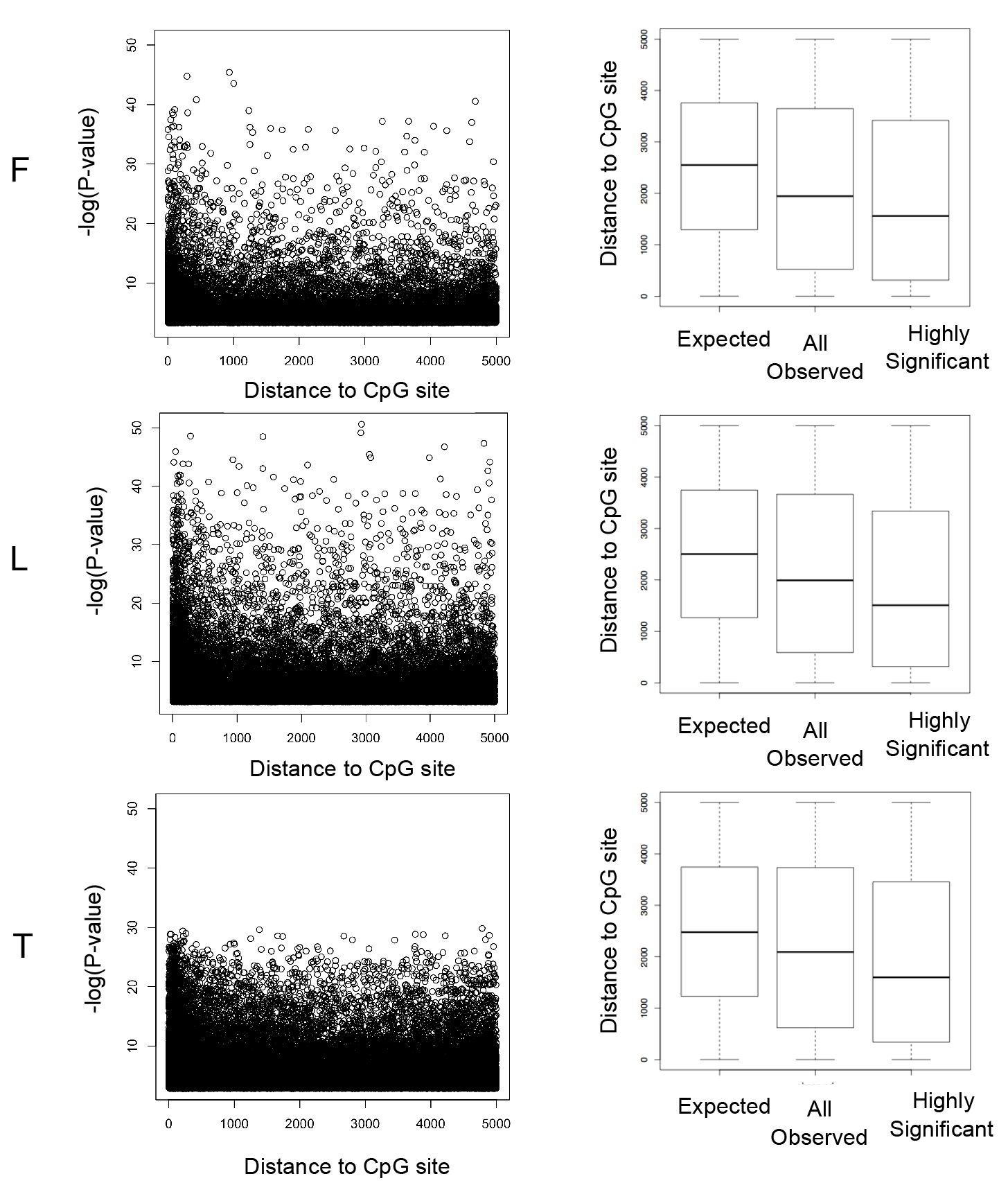

Supplement: S4 Fig — Relative distance of SNP to the CpG site (x-axis) by—log(P-value) (y-axis) in fibroblasts (F), LCLs (L) and T-cells (T; left panels). Distribution of distance to CpG site (y-axis) in expected set (random uniform), observed mQTLs at 10% FDR and highly significant mQTLs (top 25%) in each cell-type (right panels). All differences between distributions are significant, with all P < 1.3E-14 (Wilcoxon’s test). (TIF) [file pgen.1004958.s011.tif]

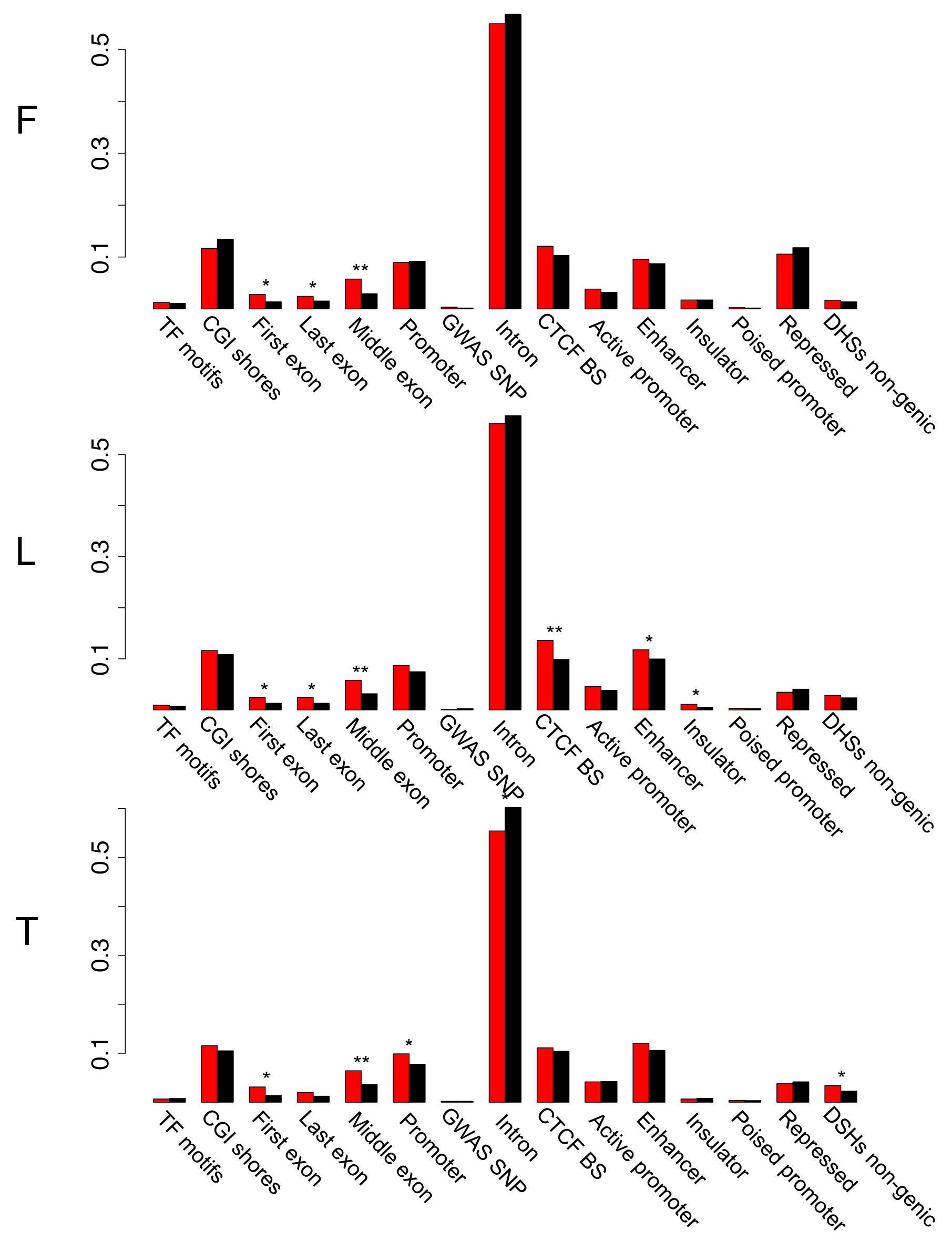

Supplement: S5 Fig — For distinct genomic regions, the proportion of overlapping eQTLs was compared to the proportion of overlapping null SNPs. One star indicates P < 0.05, two stars indicate P < 5E-04, Fisher’s exact test. Fibroblasts (F), LCLs (L), T cell (T). (TIF) [file pgen.1004958.s012.tif]

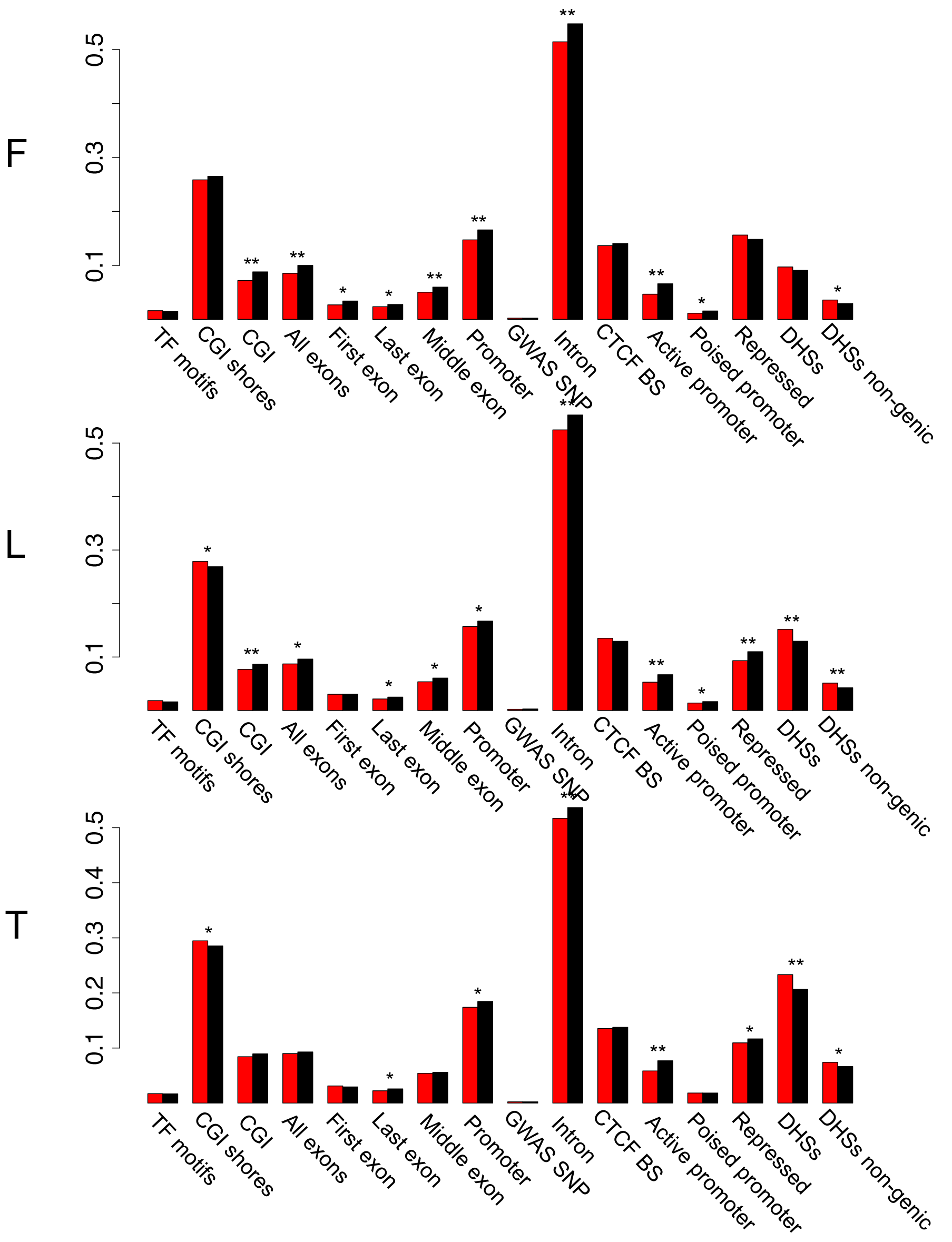

Supplement: S6 Fig — For distinct genomic regions, the proportion of overlapping mQTLs was compared to the proportion of overlapping null SNPs. One star indicates P < 0.05, two stars indicate P < 5E-04, Fisher’s exact test. Fibroblasts (F), LCLs (L), T cell (T). To note, although there is a significant depletion of mQTLs in promoters in all three cell-types, this has been shown to be different depending on whether the promoter has a CpG island or not [37]. (TIF) [file pgen.1004958.s013.tif]

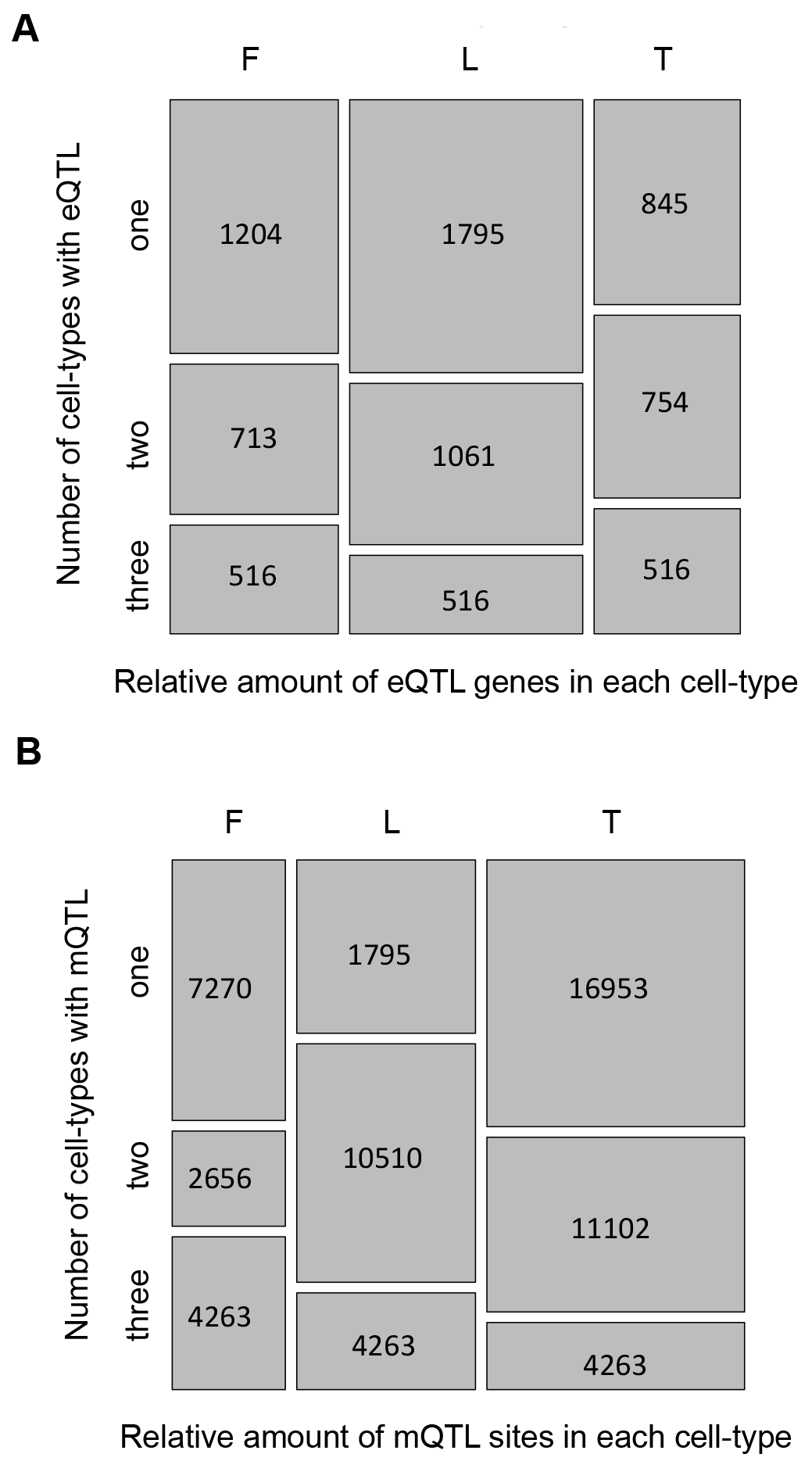

Supplement: S7 Fig — Overlap of eQTLs (A) and mQTLs (B) illustrated by the relative amount of eQTL genes or mQTL sites in each cell-type (x-axis) found significant in one, two or three cell-types (y-axis). Number of eQTL genes or mQTL sites falling in each category is indicated in the squares. (TIF) [file pgen.1004958.s014.tif]

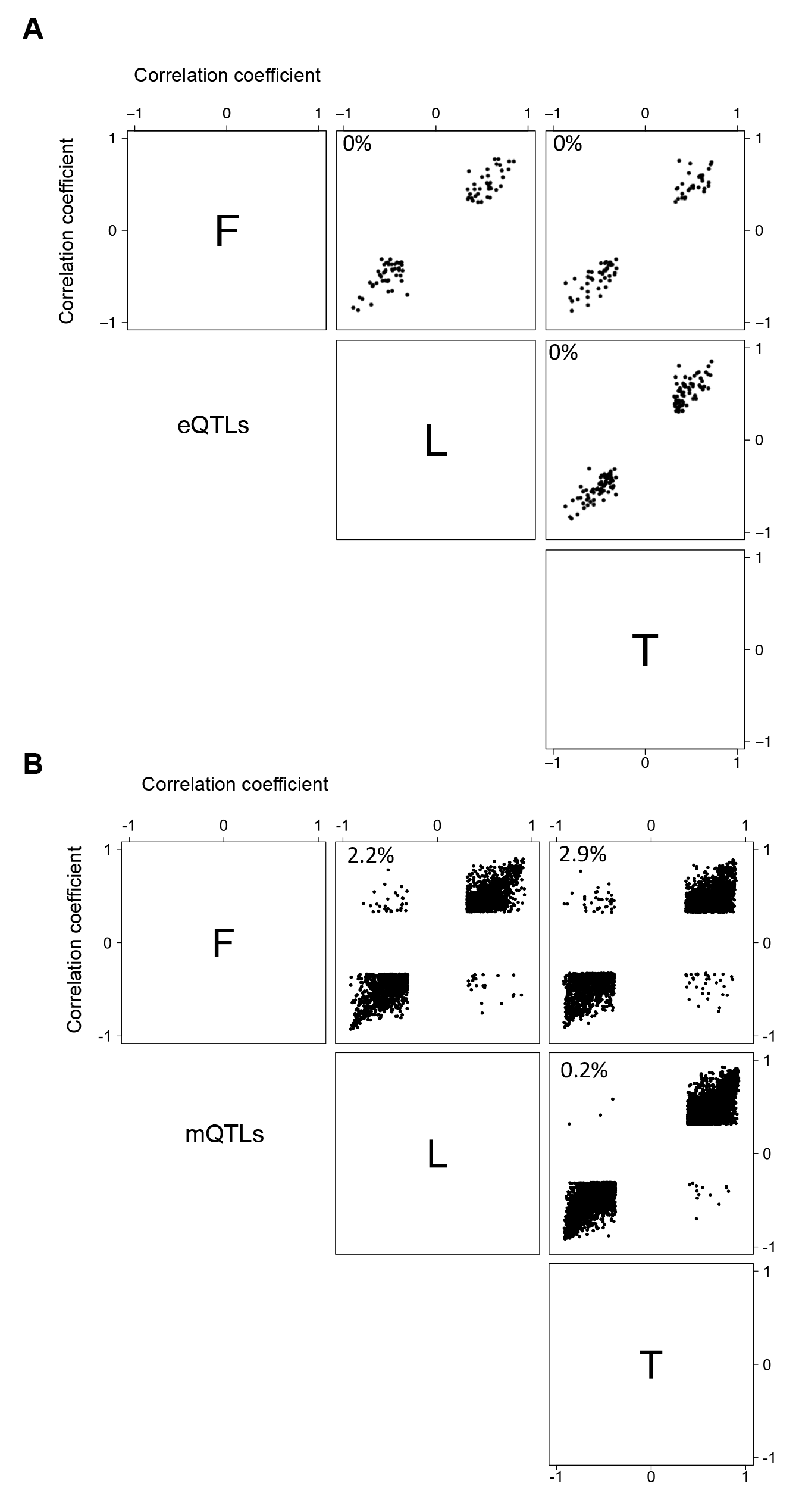

Supplement: S8 Fig — Correlation coefficients of eQTLs (A) or mQTLs (B) significant in both cell-types compared for all pair wise combinations of the three cell-types. Percentage of discordant cases (associations with opposite direction between any pair of cell-types) is indicated in the top left corner of each panel. (TIF) [file pgen.1004958.s015.tif]

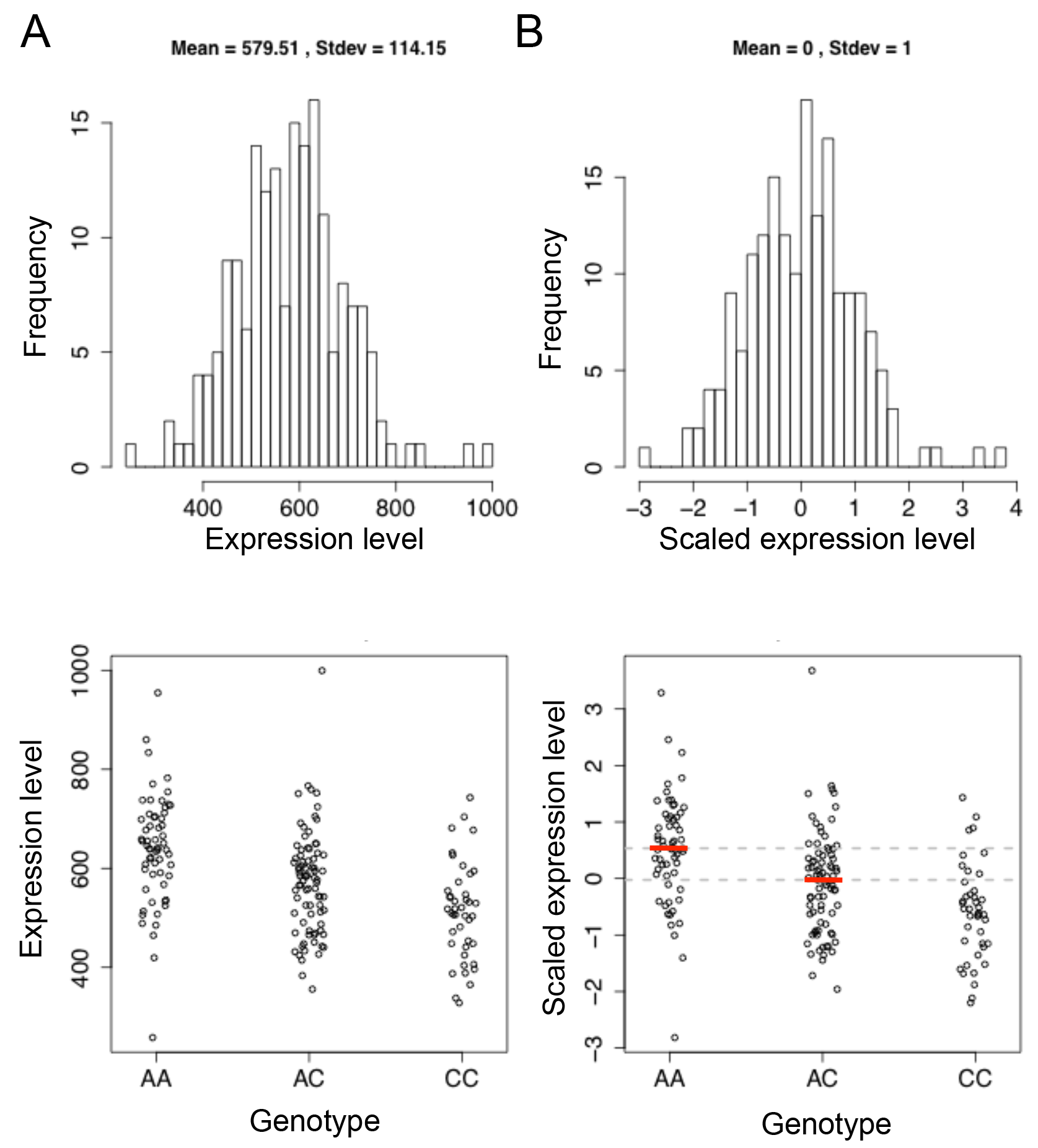

Supplement: S9 Fig — Distribution of expression levels of an exon (top) and its eQTL (bottom) for normal (A) and scaled (B) data. Scaled data is calculated by subtracting the mean and dividing by the standard deviation of normal data. Effect size of eQTLs is the difference between the median scaled expression level of the homozygous individuals for the major allele and the median scaled expression level of the heterozygous individuals (medians depicted by red lines). The effect size of this example is 0.56. (TIF) [file pgen.1004958.s016.tif]

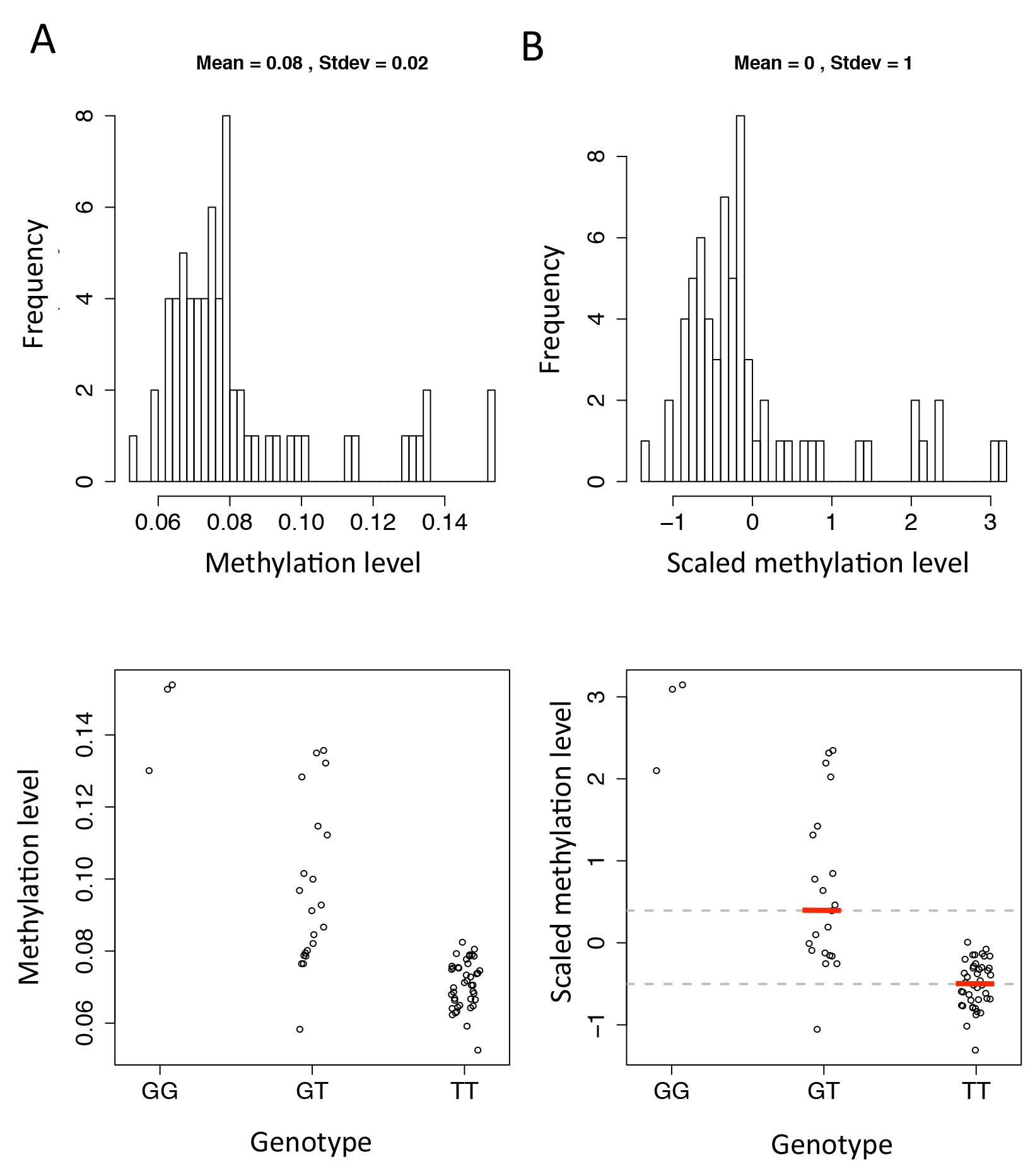

Supplement: S10 Fig — Distribution of DNA methylation levels of a CpG site (top) and its mQTL (bottom) for normal (A) and scaled (B) data. Scaled data is calculated by subtracting the mean and dividing by the standard deviation of normal data. Effect size of mQTLs is the difference between the median scaled methylation level of the homozygous individuals for the major allele and the median scaled methylation level of the heterozygous (medians depicted by red lines). The effect size of this example is −0.9. (TIF) [file pgen.1004958.s017.tif]

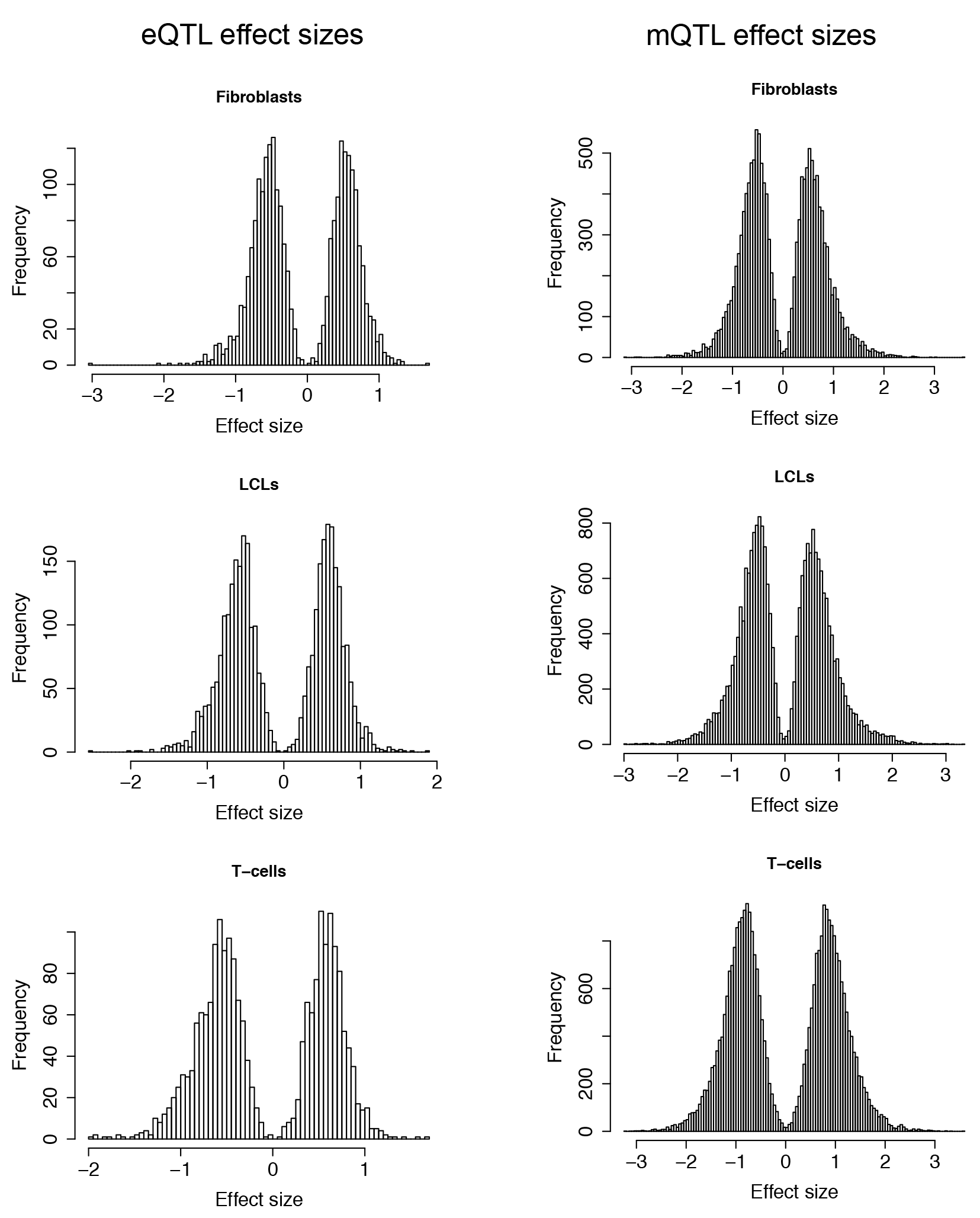

Supplement: S11 Fig — Histograms depicting the effect sizes distributions of eQTLs (left panels) and mQTLs (right panels) in each cell-type. (TIF) [file pgen.1004958.s018.tif]

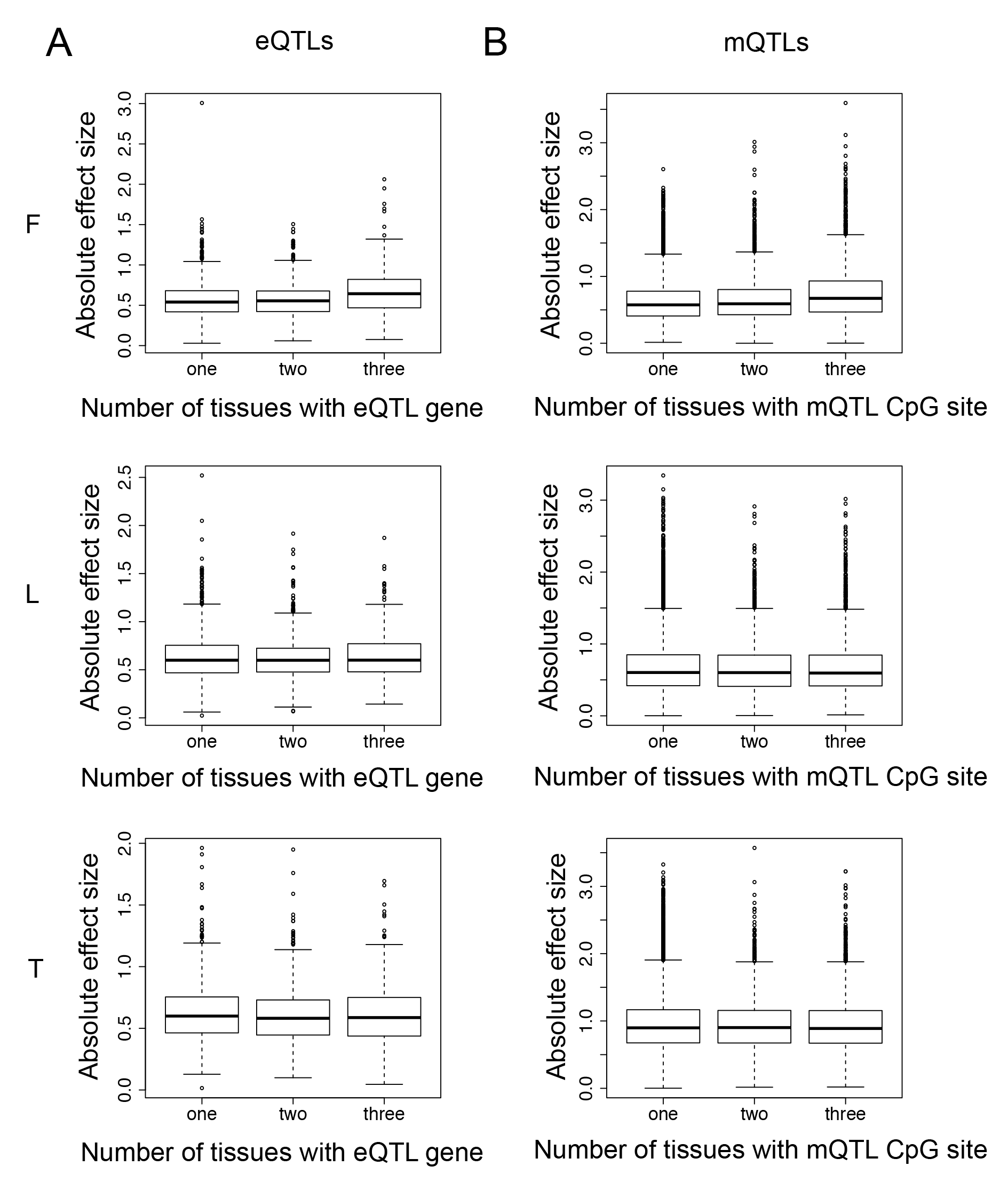

Supplement: S12 Fig — Absolute effect size distributions (y-axis) of eQTLs (A) and mQTLs (B) when the gene or CpG site has a significant eQTL or mQTL, respectively, in one, two or three cell-types (x-axis), in fibroblasts (F), LCLs (L) and T-cells (T). (TIF) [file pgen.1004958.s019.tif]

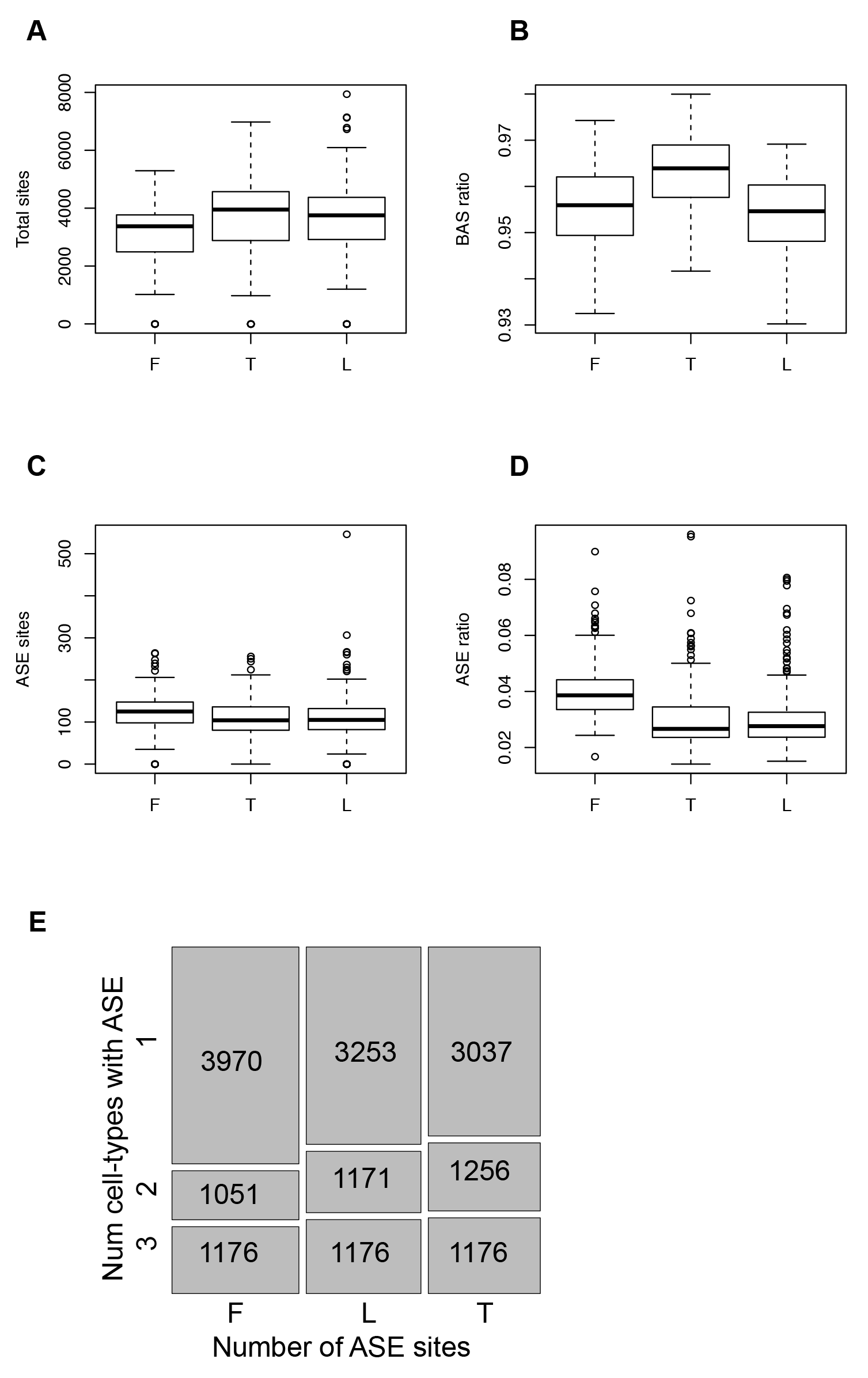

Supplement: S13 Fig — (A-E) Results of ASE analyses requiring at least 16 reads per site. (A) Distribution of number of assayed heterozygous sites per cell-type. (B) Distribution of proportion of both alleles seen (BAS) per site in each cell-type. (C) Distribution of number of allele specific expressed (ASE) sites (P < 0.005) per cell-type. (D) Distribution of percent of heterozygous sites assayed with significant ASE per sample, in each cell-type. (E) Sharing of Allele-Specific Expression (ASE) illustrated by the relative amount of ASE sites (P < 0.005) in each cell-type (x-axis) found significant in one, two or three cell-types within each individual (y-axis). Number of ASE sites falling in each category is indicated in the squares. F, L and T stand for fibroblasts, LCLs and T-cells, respectively. (TIF) [file pgen.1004958.s020.tif]

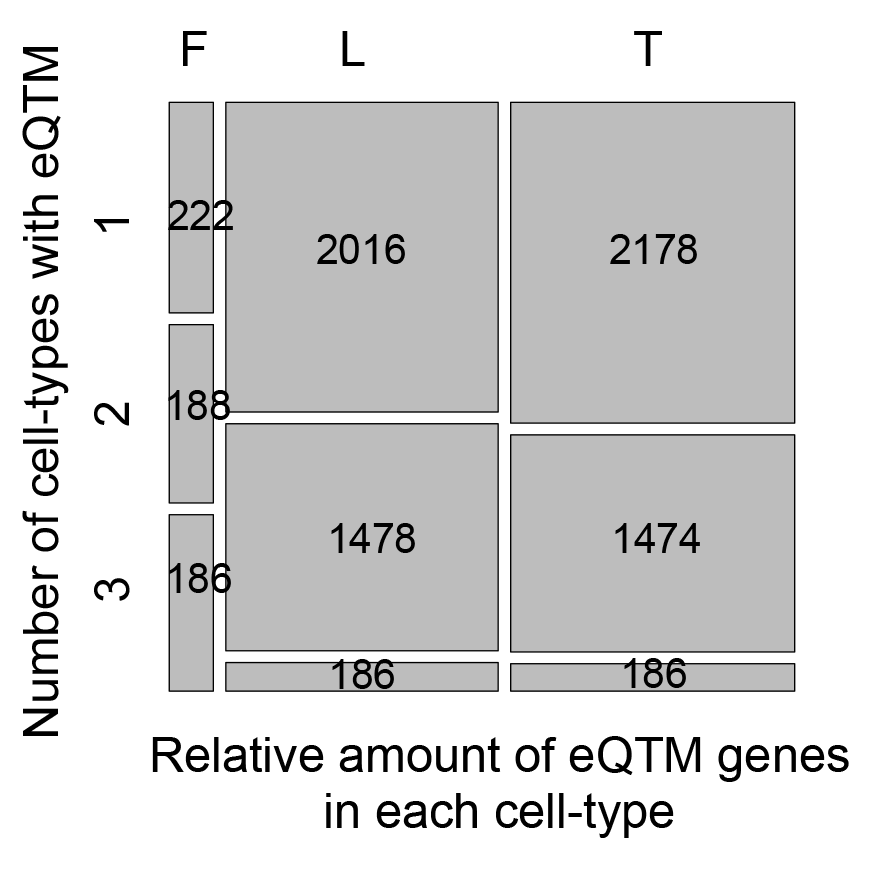

Supplement: S14 Fig — Overlap of eQTMs illustrated by the relative amount of eQTM genes in each cell-type (x-axis) found in one, two or three cell-types (y-axis). Number of eQTM genes falling in each category is indicated in the squares. 44–63% of genes with eQTMs are observed in at least two cell-types. (TIF) [file pgen.1004958.s021.tif]

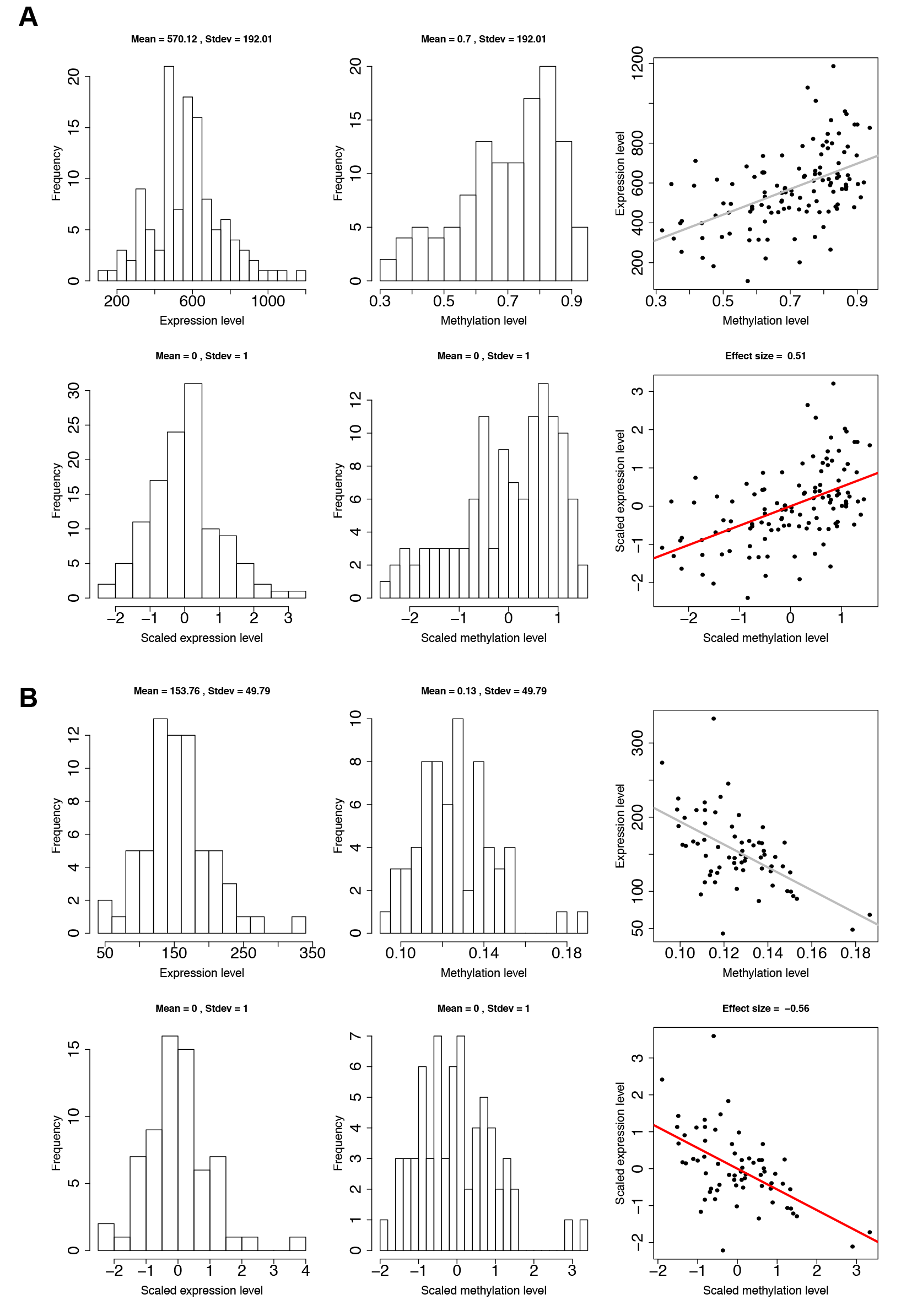

Supplement: S15 Fig — Two examples of a pos-eQTM (A) and a neg-eQTM (B) showing how effect sizes are calculated. In each (A) and (B), the top histograms show the distributions of expression level of an exon and DNA methylation levels of a CpG site that are significantly associated (top scatterplot). The bottom histograms show the same distributions for scaled data. Scaled data is calculated by subtracting the mean and dividing by the standard deviation. Effect size of eQTMs is calculated as the linear regression slope of scaled data of expression given methylation, as depicted in the bottom scatterplots on the right with the slope depicted in red and the effect size indicated above the plot. (TIF) [file pgen.1004958.s022.tif]

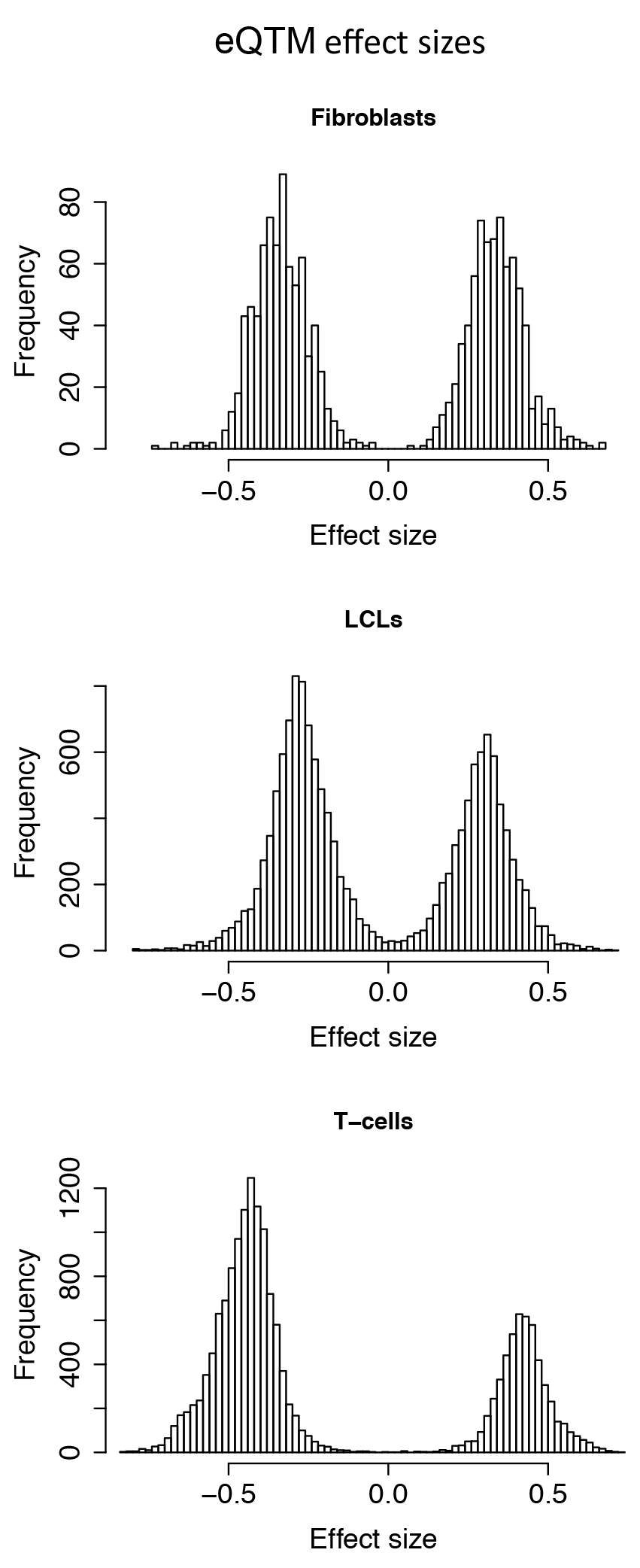

Supplement: S16 Fig — Histograms depicting the distribution of eQTM effect sizes in each cell-type. (TIF) [file pgen.1004958.s023.tif]

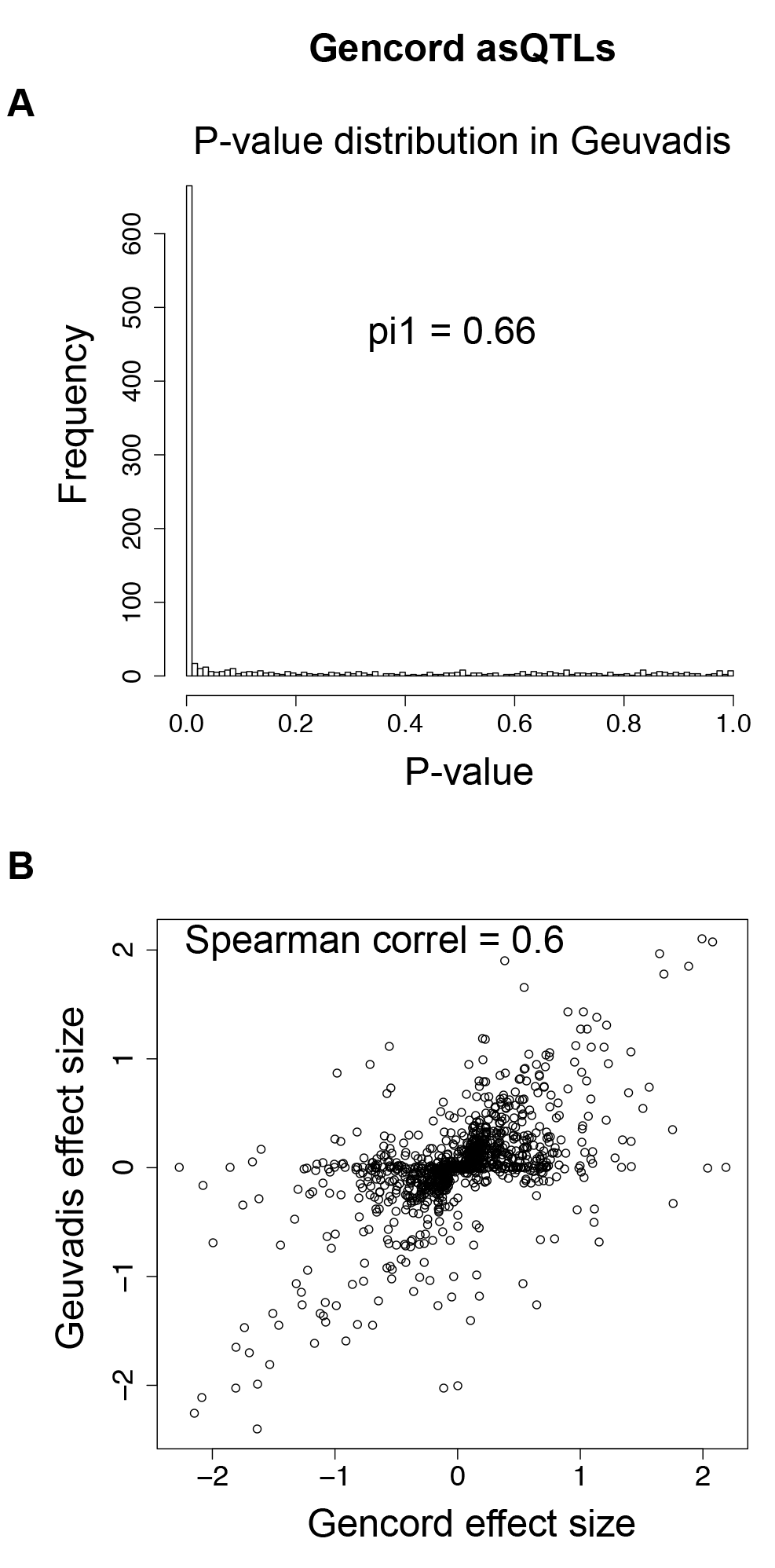

Supplement: S17 Fig — We have estimated how our LCL asQTL results replicate in an independent dataset recently published (the Geuvadis dataset, including 373 European individuals, by Lappalainen et al., 2013). (A) We have taken Gencord asQTLs and plotted the P-value distribution in Geuvadis, yielding a π1 of 0.66 (estimated fraction of true positives). (B) Plotted are the effect sizes for the Gencord asQTLs in Gencord (x-axis) and in Geuvadis (y-axis). The Spearman rank correlation coefficient between effect sizes is 0.6. (TIF) [file pgen.1004958.s024.tif]

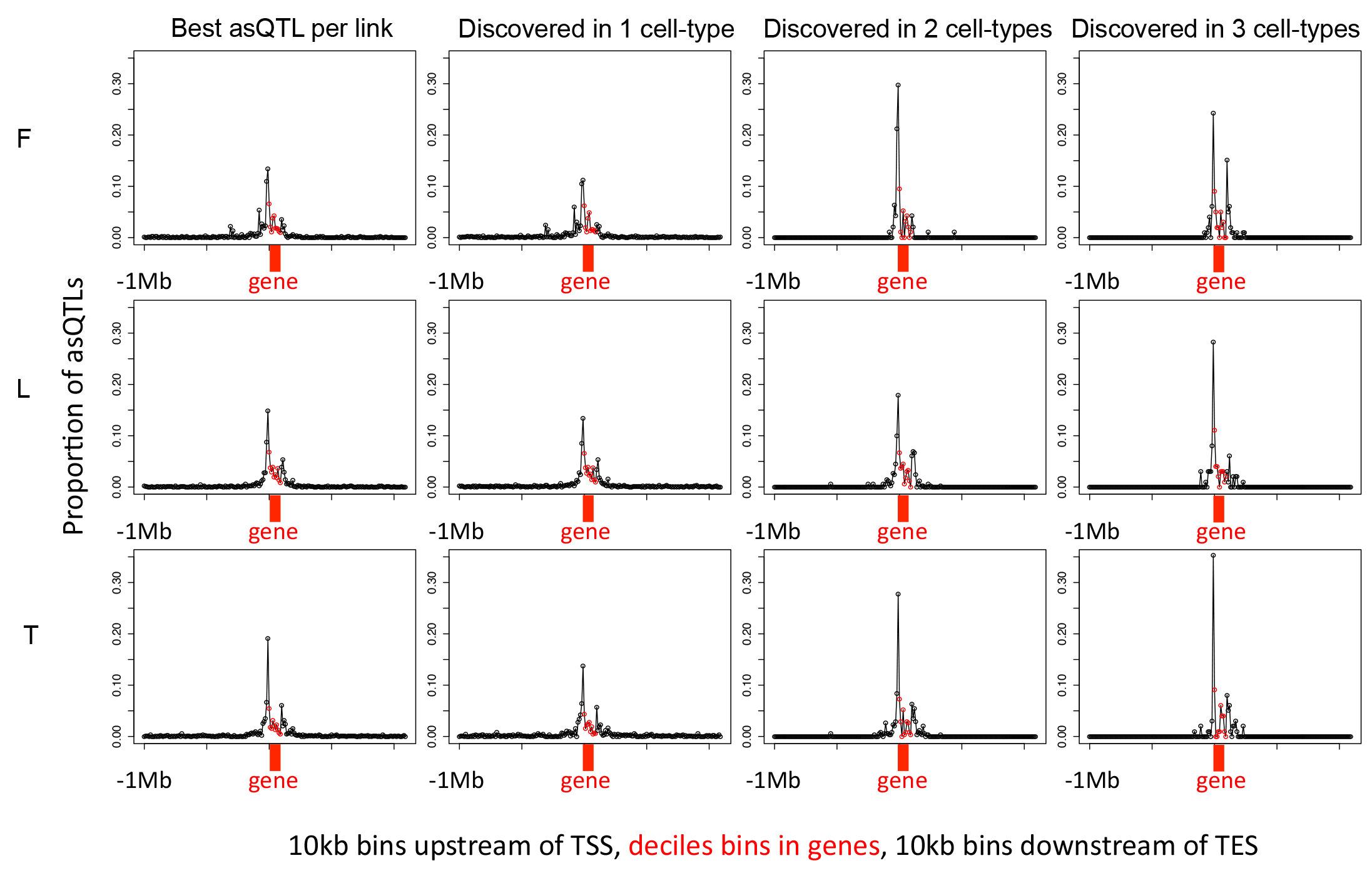

Supplement: S18 Fig — Plotted are the distributions of asQTLs (best per exon-exon link) upstream of the transcription start site (TSS), inside the gene and downstream of the transcription end site (TES) by assessing the proportion of asQTLs falling in 10kb windows for upstream of TSS or downstream of TES, and deciles inside the gene (i.e. all genes fitted to one size). We observe a high proportion of asQTLs close to the TSS and also some enrichment at the TES. (TIF) [file pgen.1004958.s025.tif]

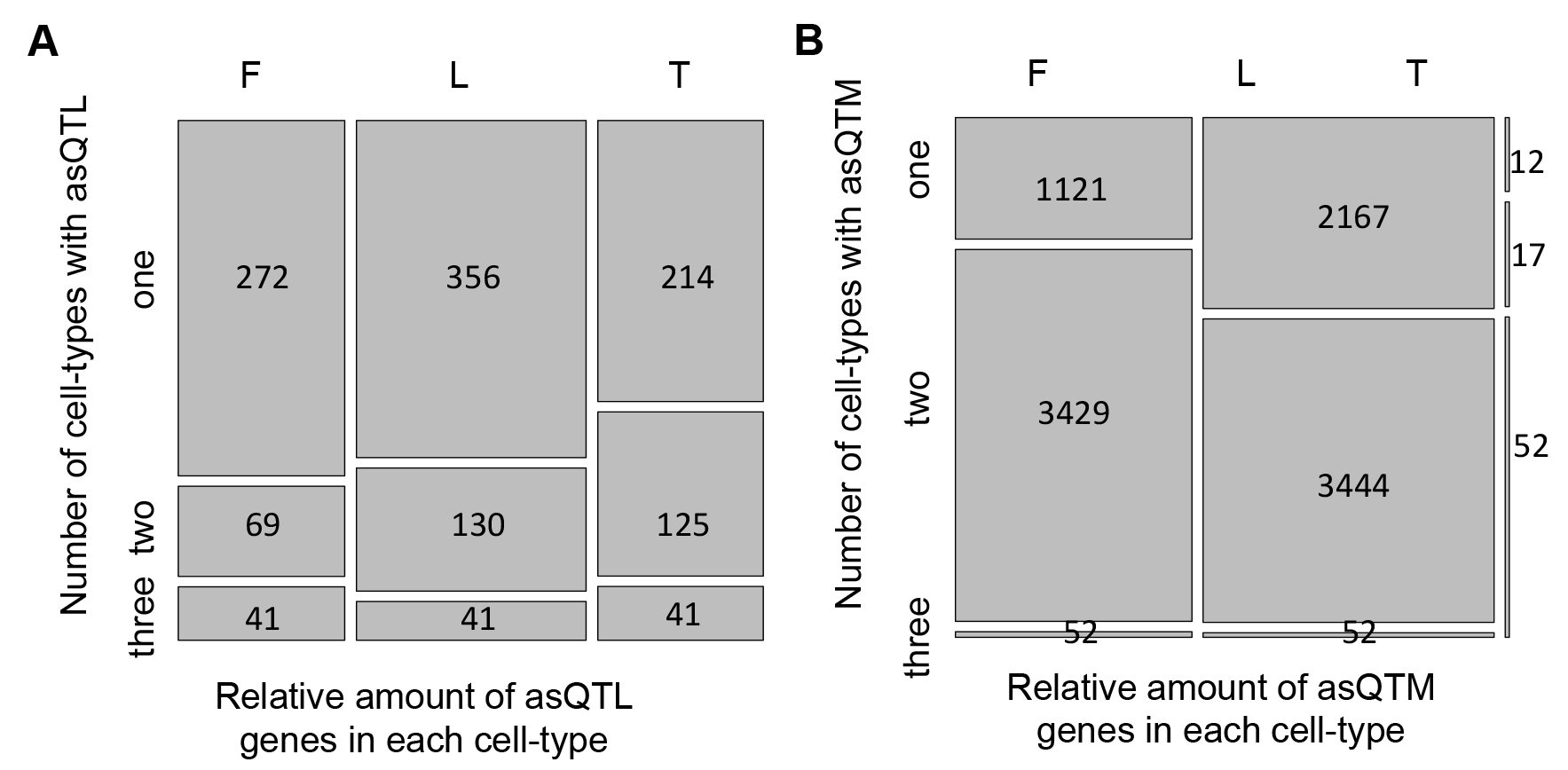

Supplement: S19 Fig — Overlap of asQTLs (A) and asQTMs (B) illustrated by the relative amount of asQTL genes or asQTM genes in each cell-type (x-axis) found significant in one, two or three cell-types (y-axis). Numbers falling in each category are indicated inside the boxes. (TIF) [file pgen.1004958.s026.tif]

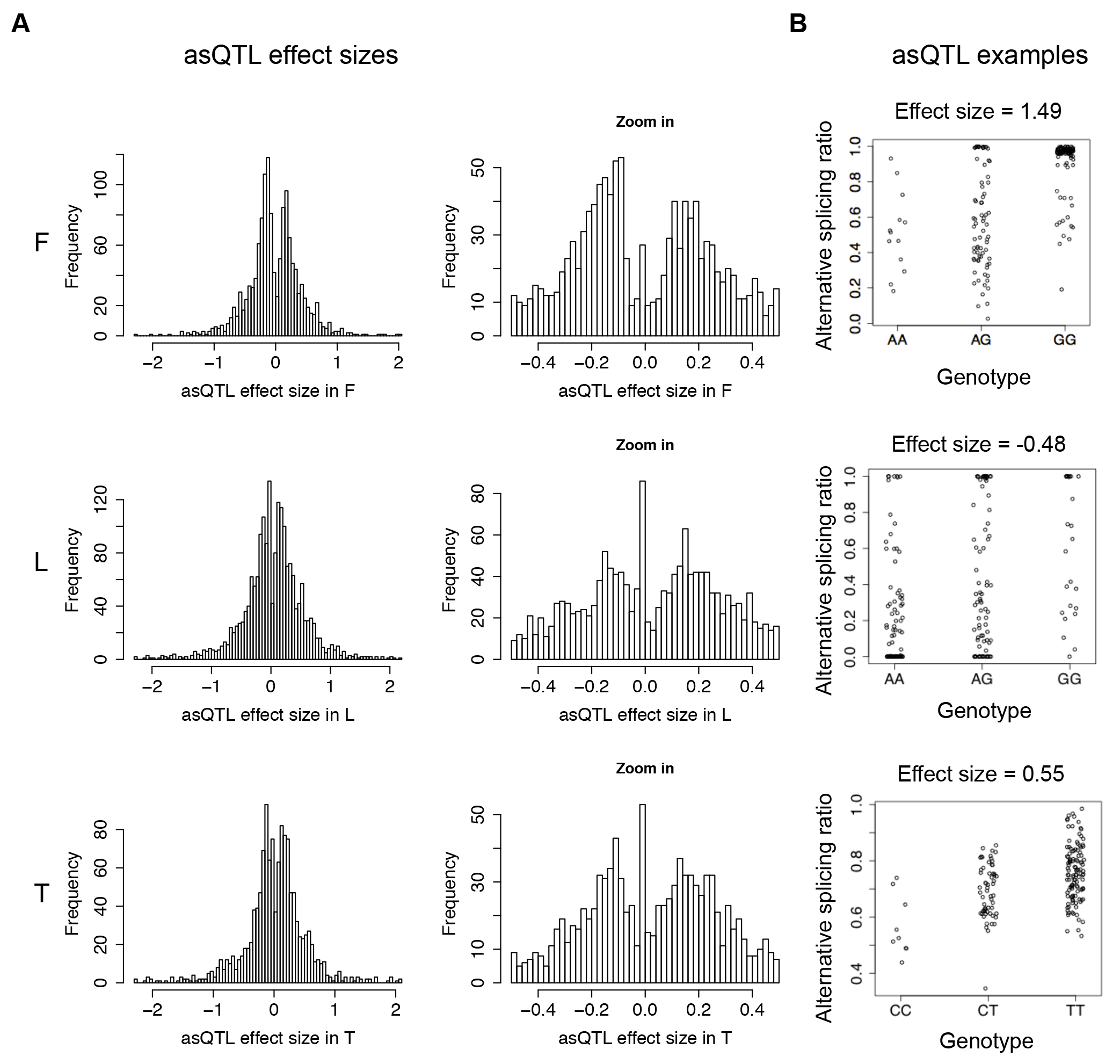

Supplement: S20 Fig — (A) Distributions of asQTL effect sizes in fibroblasts (F), LCLs (L) and T-cells (T) (left panels), with a close up vision on the center of the distributions (right panels). Effect sizes are calculated as the difference in medians of scaled alternative splicing levels between heterozygous individuals and individuals homozygous for the major allele. Scaled data is calculated by subtracting the mean and dividing by the standard deviation of normal data. (B) Plotted are examples of asQTLs with one shown for each cell-type, with the effect size indicated above each plot. (TIF) [file pgen.1004958.s027.tif]

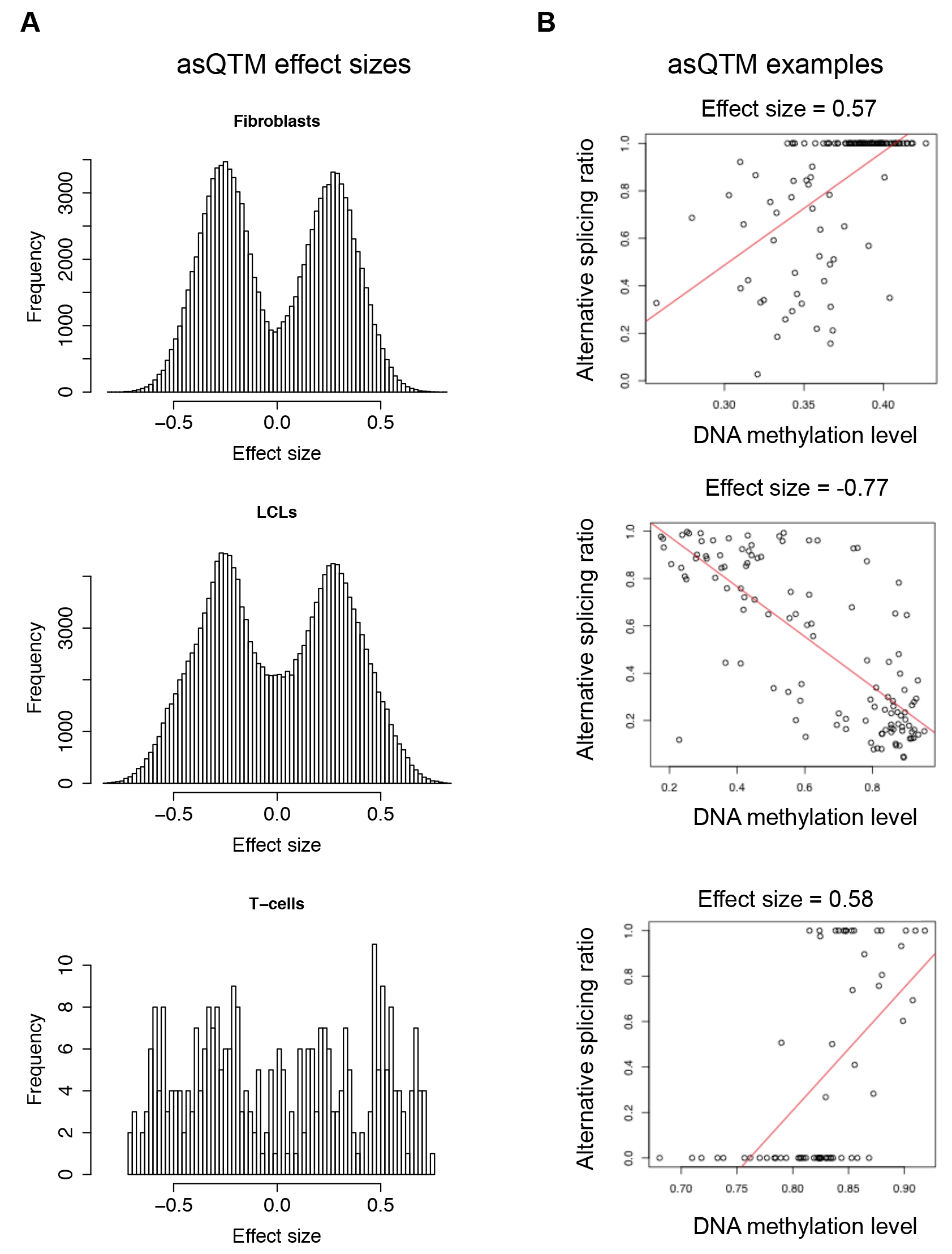

Supplement: S21 Fig — (A) Distributions of asQTM effect sizes in fibroblasts, LCLs and T-cells. (B) Plotted are examples of asQTMs in each cell-type, with the effect size indicated on the top of each plot. Effect size of asQTMs is calculated as the linear regression slope of scaled data of alternative splicing levels given DNA methylation levels, as depicted in red in the scatter plots. Scaled data is calculated by subtracting the mean and dividing by the standard deviation of normal data. (TIF) [file pgen.1004958.s028.tif]
